# A single-dose, randomized crossover study in healthy Chinese subjects to evaluate pharmacokinetics and bioequivalence of two capsules of calcium dobesilate 0.5 g under fasting and fed conditions

**DOI:** 10.1371/journal.pone.0284576

**Published:** 2023-04-21

**Authors:** Yanmei Liu, Jie Cheng, Liyu Liang, Weigang Qian, Meixian Ou, Mengqi Zhang, Yijun Wang, Yan Wang, Ka Peng, Jingying Jia

**Affiliations:** 1 Central Laboratory, Shanghai Xuhui Central Hospital, Shanghai, China; 2 Shanghai Engineering Research Center of Phase I Clinical Research & Quality Consistency Evaluation for Drugs, Shanghai, China; 3 Shanghai Zhaohui Pharmaceutical Co., Ltd., Shanghai, China; 4 Shanghai Center of Biomedicine Development, Shanghai, China; PhD, PLOS, UNITED KINGDOM

## Abstract

**Objectives:**

To compare the rate and extent of absorption of a launched generic calcium dobesilate capsule versus the branded reference formulation under fasting and fed conditions in healthy Chinese subjects, and to assess their bioequivalence and tolerability.

**Methods:**

This single-dose, open-label, randomized-sequence, 2-period crossover bioequivalence study was conducted on healthy Chinese volunteers aged 18 to 45 years. Subjects received a single 0.5 g dose of calcium dobesilate capsule under fasting or fed conditions, with a 3-day washout period between doses of the test (T) and reference (R) formulations. Blood samples were collected before and up to 24 hours after administration. The plasma concentration of calcium dobesilate was determined by a validated Liquid chromatography-tandem mass spectrometry method. Non-compartmental analysis was applied to identify the pharmacokinetic (PK) properties. The primary PK parameters including the maximal plasma concentration (C_max_), the area under the plasma concentration-time curve (AUC_0-t_), and the AUC extrapolated to infinity (AUC_0-inf_) were used for bioequivalence evaluation.

**Results:**

The mean of PK parameters for T and R capsules under fasting (fed) condition were: C_max_, 13.57 (6.71) and 12.59 (7.25) μg/mL; AUC_0-t_, 97.32 (79.74) and 96.97 (80.71) h*μg/mL; AUC_0-inf_, 101.68 (88.01) and 101.64 (87.81) h*μg/mL. The 90% confidence intervals (CIs) of GMRs under fasting (fed) condition were: C_max_, 97.91%-116.62% (88.63%-96.53%); AUC_0-t_, 97.15%-104.00% (96.58%-101.39%); and AUC_0-inf_, 97.19%-102.89% (98.67%-103.99%). These 90% CIs were all within the bioequivalence range of 80%-125%. All adverse events were mild.

**Conclusion:**

In this study, the T calcium dobesilate 0.5 g capsule was bioequivalent to the reference product under both fasting and fed conditions. Taking food would slow down its rate and reduce its amount of absorption. Both formulations were generally well tolerated.

## Introduction

Calcium dobesilate is a kind of vasodilator selectively acting on the capillary wall, which can not only adjust and improve the permeability and brittleness of the capillary wall but also inhibit bradykinin and other active substances, mainly used for the treatment of capillary diseases caused by a variety of reasons [1, 2]. After single oral administration of 0.5 g calcium dobesilate, the plasma concentration was above 6 μg/mL between the 3rd hour and the 10th hour, and reached the maximum (C_max_) at 6 hours (T_max_), averaging 8 μg/mL [2]. The plasma concentration was about 3 μg/mL 24 hours after administration. The protein binding rate is 20%-25%. Trace amounts are present in breast milk. Calcium dobesilate does not enter the enterohepatic circulation and is mainly excreted in its original form, with only 10% excreted as metabolites. Within 24 hours of administration, approximately 50% of the oral dose is excreted from the urine and approximately 50% is excreted from the stool. The plasma half-life was about 5 hours [3].

Calcium dobesilate is often used in clinical treatment for some diabetic complications, such as diabetic retinopathy and diabetic nephropathy [4–6], with evidence of obvious improvement in clinical efficacy when using a combination of calcium dobesilate. Launched in China in 2001, calcium dobesilate has been proven to be effective, safe, and reliable after several years of clinical application [7, 8], which is worthy of clinical promotion.

Although the branded drug of calcium dobesilate (Doxium) has been on the market for many years, it is still high-cost for some patients. Therefore, the approved generic product of calcium dobesilate is needed to support the marketing application and help relieve the medical cost burdens. Bioequivalence studies comparing generic to innovator products are required for marketing a new generic product by the National Medical Products Administration (NMPA) of China [9]. If the rate and extent of absorption of the generic drug and the reference drug do not show any significant difference in bioequivalence (BE) study in human subjects, the generic drug is considered to be bioequivalent to the reference drug [10].

Pharmacokinetic (PK) properties and bioequivalence of calcium dobesilate in different dosage forms by different manufacturers have been studied before, including in Chinese subjects [11–14]. For example, a study of dietary influences on the pharmacokinetics of calcium dobesilate in 8 healthy Chinese male volunteers (single-dose, randomized, open, 3-cycle cross-trial design) showed that eating delayed the absorption of calcium dobesilate capsules in healthy volunteers, with no serious adverse events reported [12]. Although relevant studies have been reported, the number of studies is small, and the sample size is not very sufficient. Besides, there hasn’t been enough post-meal research available confirming the effects of food on the PK of calcium dobesilate. Therefore, this study aims to compare the bioequivalence of a launched domestic calcium dobesilate capsule (0.5 g, the test preparation) and the branded calcium dobesilate capsule (0.5 g, the reference preparation Doxium^®^) under fasting and fed conditions in healthy Chinese volunteers, to supplement the data on the PK and dietary effects of calcium dobesilate in Chinese population, to meet the needs of post-market evaluation of generic drug in China [15], and to meet the requirement of New Drug Application.

## Materials and methods

### Subjects

This study was conducted at the Phase I clinical research center of Shanghai Xuhui Central Hospital. All subjects had been informed and provided signed written informed consent before participating in the study. Subjects of the fasting study were recruited in July 2018, and subjects of the fed study were recruited in October 2018. The investigators in the clinical center were required to comply with the rules that protect the subject’s privacy. Except for them, nobody had access to information that could identify individual participants during or after data collection.

The inclusion criteria comprised healthy Chinese volunteers aged 18 to 40 years, with a body mass index between 19.0 to 26.0 kg/m^2^, in good health and physical condition as determined by medical history, vital signs, physical examinations, 12-lead ECG, chest X-ray examination and laboratory tests (blood chemistry, hematology, urinalysis, hepatitis B surface antigen, hepatitis C antibody, HIV antibody, and syphilis antibody).

The exclusion criteria included a history of heavy smoking (more than 10 cigarettes per day) or suspicion of drug dependence and/or alcohol abuse. Those who had participated in other clinical trials or donated or lost blood of more than 400 mL within 3 months were excluded. Subjects were instructed from taking any medications for at least 14 days before study drug administration.

### Study designs

This study was a single-dose, open-label, randomized-sequence, 2-period crossover bioequivalence study. The study (Trial registration number in http://www.chinadrugtrials.org.cn/: CTR20181854; ChiCTR registry number in http://www.chictr.org.cn/: ChiCTR2200067026) was conducted in accordance with the Declaration of Helsinki [16], the Guidelines for Good Clinical Practice of the International Conference on Harmonisation [17], and the Guidelines for Good Clinical Principles recommended by NMPA of China [18]. The study protocol and informed-consent form was approved by the ethic committee of Shanghai Xuhui Central Hospital. According to NMPA guidelines on the investigation of bioequivalence [19], it was recommended that for orally-administered preparations, bioequivalence studies under fasting and fed conditions are usually required.

Subjects were randomly assigned to enter TR or RT administration sequence according to the randomized table generated by SAS version 9.4 (SAS Institute Inc., Cary, North Carolina) after being admitted to the hospital. The sample size for fasting and fed conditions was 26 and 72. It was calculated based on the results of preliminary studies by using PASS (Version 11.0.7) software. They fasted for at least 10 hours and were administered the test (T, lot # BR171203, expiration date 11/2019, Shanghai Zhaohui Pharmaceutical Co., Ltd.) or the reference (R, lot #GK8660, expiration date 12/2018, Ebewe Pharma Ges.m.b.H. Nfg.KG) product of calcium dobesilate Capsules according to their administration sequences in the next day. After a 3-day wash-out, the alternate treatment was administered in the second period. The fasting study was conducted from July 5, 2018, to July 19, 2018. The fed study was conducted from October 10, 2018, to December 3, 2018.

Subjects who participated in the fed study must take a high-fat, high-calorie breakfast (about 913 kcal, including about 152 kcal protein, 263 kcal carbohydrate, and 499 kcal fat) 30 minutes before dosing and finish it within 30 minutes. Study drugs were administered with 240 mL of water under the supervision of a qualified pharmacist. Subjects were not allowed to drink water 1 hour before or after dosing, except for the water used for drug administration. Food intake was strictly controlled during the study period, any food or drink other than a uniform diet was prohibited. Standardized lunch and dinner were provided approximately 4 and 10 hours postdose, respectively.

All volunteers who participated were under medical supervision by a physician throughout the study. 2 mL of blood samples were drawn into a vacuum blood collection tube containing heparin sodium. Samples were obtained at 0 (pre-dosing), 1, 2, 3, 3.5, 4, 4.5, 5, 5.5, 6, 7, 8, 10, 12, 14, and 24 hours after dosing. The sample collection in the fasting study was during July 14–15 and July 17–18, 2018. The sample collection in the fed study was during October 16–17, 19–20, 23–24, and 26–27, 2018. After sample collection, plasma was separated by centrifugation (1500 g ×10 minutes, 4°C), transferred into a polypropylene tube within 1 hour, and stored at -80°C until analyzed by LC-MS/MS method.

### Tolerability assessments

Tolerability assessments included physical examinations, vital signs (oral body temperature, pulse rate, and sitting blood pressure), 12-lead ECGs, clinical laboratory tests, and adverse events (AEs) monitoring.

### Determination of plasma calcium dobesilate concentrations

The concentration of calcium dobesilate in plasma was determined by a validated LC-MS/MS method [20, 21] with Deuterium 3 labeled calcium dobesilate (CaD-D3) as the internal standard (IS).

The bioanalytical method and validation results are briefly described as follows: an aliquot (10 μL) of IS working solution (10 μg/mL CaD-D3-10% methanol-water solution) was added to 100 μL of plasma and mixed well, then 400 μL of acetonitrile was added into each sample and mixed well (vortex 30 s at 1400 rpm). The mixture was transferred to a dephosphorized sheet and made pressure filtration by Positive Pressure-96 Processor. The filtrate was diluted 10 times with water and mixed for 30 s at 2000 rpm. Then the filtrate was injected into the chromatographic system for analysis.

Chromatographic conditions were as follows: The column was Luna^®^Omega PS C18 100A (2.1×50 mm, 1.6 μm, Phenomenex corporation, USA, lot B17121), and the column temperature was 40°C, with a flow rate of 0.6 mL/min. Mobile phase A was an aqueous solution containing 0.05% formic acid and 1 mM ammonium acetate, and the Mobile phase B was an acetonitrile solution containing 0.05% formic acid.

Calcium dobesilate and the IS were quantitated by multiple reaction monitoring (MRM) mode with negative electrospray ionization. The detection ions of calcium dobesilate and the internal standard were 188.9→79.9 amu and 191.9→79.9 amu, respectively.

There was no impurity peak in blank plasma affecting the determination accuracy. Under the selected LC-MS/MS conditions, the retention time of calcium dobesilate and the internal standard was stable, and the response could be reproduced. The linear range of calcium dobesilate is 0.2–20 μg/mL, and the lower limit of quantitation is 0.2 μg/mL. The ratio of concentration and peak area has a good linear relationship.

The developed method was validated for selectivity, linearity, accuracy, precision, sensitivity, recovery, reproducibility, and stability according to the guidance for bioanalytical method validation of FDA/CFDA/EMA.

### Pharmacokinetic and statistical analysis

PK parameters were calculated with a non-compartmental model using Phoenix WinNonlin7.0 and other statistics were finished using SAS V9.4 [22]. Descriptive statistics including mean, standard deviation (SD), median, maximum, minimum, geometric mean, and coefficient of variation (CV) were used to summarize the PK data of time-matched concentrations and parameters. The graphs of individual C-T curve and mean C-T curve were plotted. C_max_ and T_max_ were obtained directly from the C-T curves of calcium dobesilate. AUC_0−t_ was calculated according to the linear trapezoidal rule. AUC_0−inf_ was calculated as AUC_0−t_ + C_t_/λ_z_, where C_t_ was the concentration at the last available point and λ_z_ was the slope of the linear regression of the log-transformed C-T curve. Calcium dobesilate plasma t_1/2_ was calculated as 0.693/λ_z_.

The major PK parameters used to evaluate the bioequivalence of these two preparations were C_max_, AUC_0-t_, and AUC_0-inf_. ANOVA was performed on the natural logarithm (ln)-transformed major PK parameters. In the ANOVA model, sequence, treatment, and period are fixed effects, and subjects (sequence) are random effects. If the 90% CIs of test/reference geometric mean ratio (GMR) for major PK parameters were located within 80%-125% both under fasting and fed conditions, the two preparations would be considered bioequivalent [23].

## Results

### Demographic data

**Fig 1:**
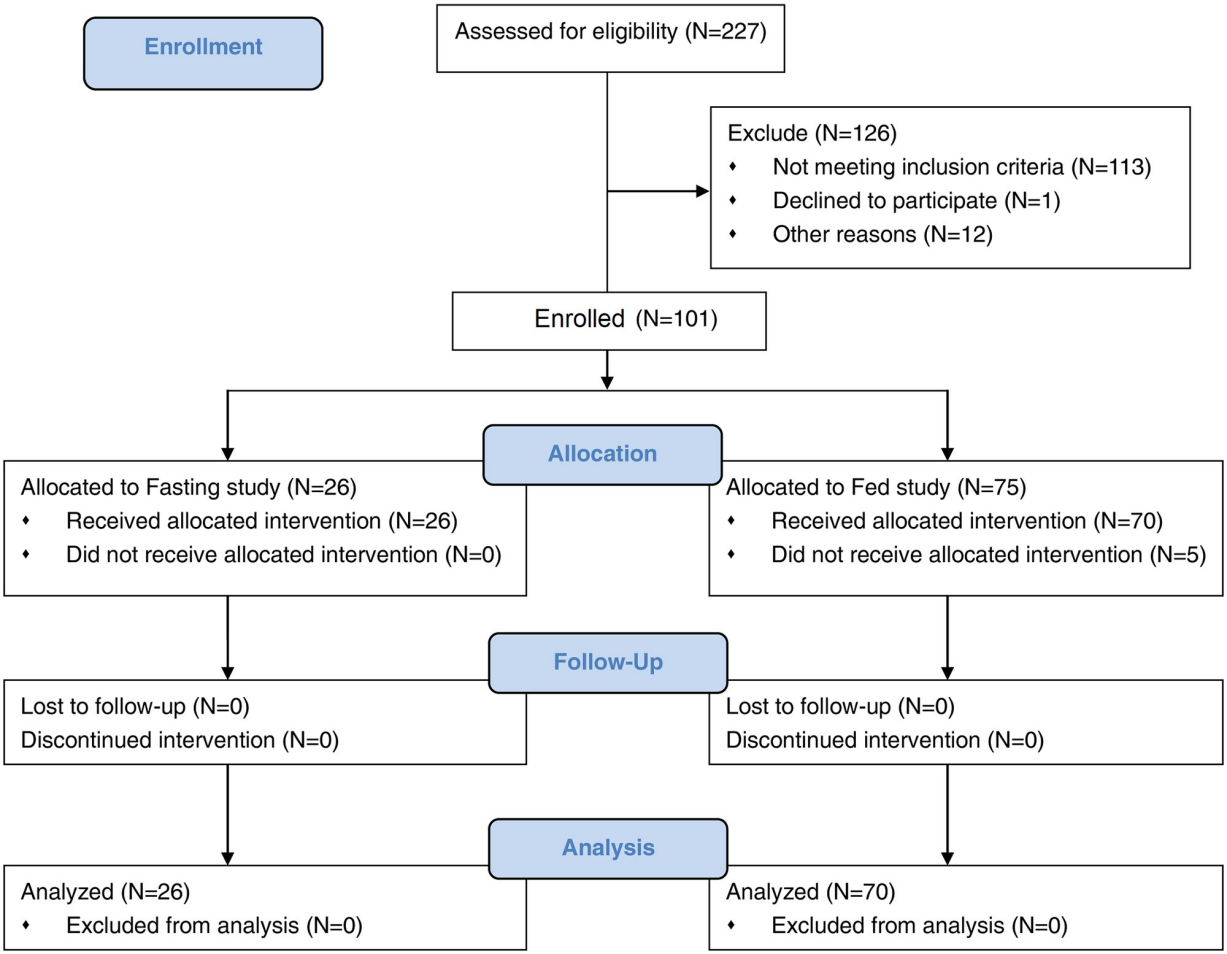
Study flow diagram.

A total of 101 (26 in the fasting study and 75 in the fed study) healthy Chinese volunteers were enrolled but 5 subjects in the fed study dropped out before drug administration (Fig 1). The remaining 96 subjects all completed the study. Due to a large number of subjects, the fed study was conducted in two batches (36 for the first batch and 34 for the second batch). There were 20 (76.9%) males and 6 (23.1%) females in the fasting study, and 65 (92.9%) males and 5 (7.1%) females in the fed study. The demographic characteristics of these volunteers are summarized in Table 1.

**Table 1:** Demographic characteristics of the healthy Chinese volunteers in fasting and fed study.

Demographics	Fasting (N = 26)			Fed (N = 70)		
	Mean±SD	Median	Min, Max	Mean±SD	Median	Min, Max
Age (years)	26.7±5.05	26.5	19, 37	25.9±4.65	25.0	19, 35
Height (cm)	164.98±7.878	166.00	150, 177	168.73±6.394	168.00	156, 187
Weight (kg)	61.29±8.011	62.20	45.4, 74.5	64.71±6.620	65.00	51, 80.5
BMI (kg/m^2^)	22.46±1.784	22.10	19.2, 25.2	22.73±1.995	22.50	19, 26

### Tolerability assessments

All subjects with drug-related AEs for the test and reference calcium dobesilate capsules under fasting conditions were reported by 0 and 1 (3.8%), respectively, while under fed conditions, drug-related AEs for test and reference were 4 (5.7%) and 9 (12.9%), respectively (Table 2). The most common drug-related AE in the test product was hypotension, and in the reference product was diarrhea. All AEs were relatively transient and were considered to be mild intensity (grade one) by the investigators. There was no serious AE reported, and none of the subjects withdrew from the study due to AE. No new clinically significant abnormalities on physical examinations, vital sign measurements, and ECG recordings were observed after administration.

**Table 2:** All drug-related AEs of test and reference products of calcium dobesilate capsules after single-dose oral administration under fasting and fed conditions in healthy Chinese volunteers.

Drug-related AEs	Number (%) of subjects with AEs			
	Fasting (N = 26)		Fed (N = 70)	
	Test	Reference	Test	Reference
Positive urinary erythrocyte	0 (0.0)	1 (3.8)	1 (1.4)	4 (5.7)
Positive urinary leukocyte	0 (0.0)	1 (3.8)	0 (0.0)	0 (0.0)
Increase in ALT	0 (0.0)	0 (0.0)	1 (1.4)	3 (4.3)
Increase in hemobilirubin	0 (0.0)	0 (0.0)	1 (1.4)	2 (2.9)
Bellyache	0 (0.0)	0 (0.0)	1 (1.4)	0 (0.0)
Increase in defecation frequency	0 (0.0)	0 (0.0)	1 (1.4)	0 (0.0)
**A total of subjects with AEs**	**0 (0.0)**	**1 (3.8)**	**4 (5.7)**	**9 (12.9)**

### Pharmacokinetic analysis

**Fig 2:**
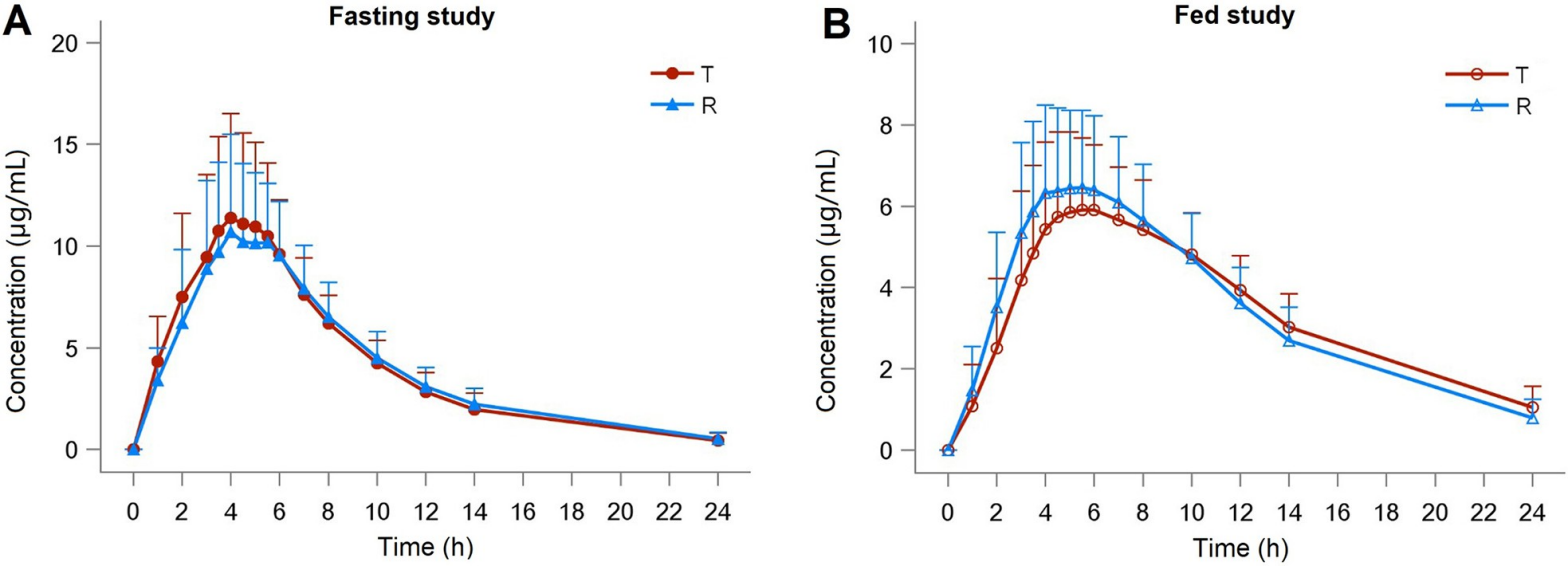
Mean (SD) plasma concentration-time curves for the test and reference of calcium dobesilate capsules 0.5 g after oral administration under fasting (N = 26) and fed (N = 70) conditions in healthy Chinese volunteers.

The mean (±SD) plasma C-T curves of calcium dobesilate after single-dose administration of test or reference capsules (0.5 g) under fasting and fed conditions are shown in Fig 2. The primary PK parameters under fasting and fed conditions are listed in Table 3.

**Table 3:** Pharmacokinetic properties of test and reference products of calcium dobesilate capsules after single-dose oral administration in healthy Chinese volunteers under fasting and fed conditions.

PK Parameters	Statistics	Fasting (N = 26)		Fed (N = 70)	
		Test	Reference	Test	Reference
C_max_ (μg/mL)	Mean±SD	13.57±4.39	12.59±3.77	6.71±1.74	7.25±1.89
	CV%	32.4	29.9	25.9	26.1
AUC_0-t_ (h*μg/mL)	Mean±SD	97.32±14.94	96.97±15.83	79.74±12.67	80.71±13.92
	CV%	15.3	16.3	15.9	17.2
AUC_0-inf_ (h*μg/mL)	Mean±SD	101.68±15.47	101.64±15.10	88.01±13.61	87.81±15.04
	CV%	15.2	14.9	15.5	17.1
t_1/2_ (h)	Mean±SD	4.34±1.43	4.78±1.33	5.89±1.22	5.31±1.48
	CV%	33.0	27.9	20.8	27.8
λ_z_ (1/h)	Mean±SD	1.75±0.52	1.57±0.45	1.23±0.29	1.42±0.44
	CV%	29.8	29.0	23.7	30.9
T_max_ (h)	Median (Min, Max)	4.00 (2, 7)	4.25 (2, 6)	5.50 (3, 14)	5.00 (3, 14)
	CV%	33.5	26.3	35.1	36.3

In the fasting study, the mean±SD values of C_max_ for the test and the reference products were 13.57±4.39 and 12.59±3.77μg/mL, respectively. The mean±SD values of AUC_0-t_ and AUC_0-inf_, were 97.32±14.94, 101.68±15.47 h*μg/mL for the test product, and 96.97±15.83, 101.64±15.10 h*μg/mL for the reference product. The median (min, max) of T_max_ for the test and reference products were 4.00 (2, 7) and 4.25 (2, 6) hours, respectively. The mean ± SD values of t_1/2_ for the test and reference products were 4.34±1.43 and 4.78±1.33 hours, respectively.

In the fed study, the mean ± SD values of PK parameters for the test and the reference products were as follows: C_max_, 6.71±1.74 and 7.25±1.89 μg/mL; T_max_ median (min, max), 5.50 (3, 14) and 5.00 (3, 14) hours. AUC_0–t_, 79.74±12.67 and 80.71±13.92 h*μg/mL. AUC_0-inf_, 88.01±13.61 and 87.81±15.04 h*μg/mL. And t_1/2_, 5.89±1.22 and 5.31±1.48 hours, respectively.

The 90% Cls of the ratio of test to reference for the ln-transformed C_max_, AUC_0-t_, and AUC_0-inf_ are shown in Table 4. In the fasting study, the 90% CIs of the ratios for C_max_, AUC_0-t_, and AUC_0-inf_ were 97.91% to 116.62%, 97.15% to 104.00%, and 97.19% to 102.89%, respectively. In the fed study, the 90% CIs of the ratios for C_max_, AUC_0–t_, and AUC_0−inf_ were 88.63% to 96.53%, 96.58% to 101.39%, and 98.67% to 103.99%, respectively. The 90% CIs of the ratios for major PK parameters all fall within the range of 80.00% to 125.00%, indicating that the predetermined criteria for bioequivalence were met.

**Table 4:** Bioequivalence evaluation of test and reference products of calcium dobesilate capsules in healthy Chinese volunteers under fasting and fed conditions.

	TestGeoLSM	RefGeoLSM	Ratio (T/R) %	90% CI	CV_w_ %
**Fasting Study (N = 26)**					
C_max_ (μg/mL)	12.89	12.06	106.85	97.91–116.62	18.58
AUC_0-t_ (h*μg/mL)	96.22	95.73	100.52	97.15–104.00	7.19
AUC_0-inf_ (h*μg/mL)	100.55	100.55	100.00	97.19–102.89	6.01
**Fed Study (N = 70)**					
C_max_ (μg/mL)	6.51	7.03	92.50	88.63–96.53	15.23
AUC_0-t_ (h*μg/mL)	78.75	79.58	98.96	96.58–101.39	8.63
AUC_0-inf_ (h*μg/mL)	87.70	86.58	101.29	98.67–103.99	8.72

GeoLSM, Geometric least squares mean; CV_w_, within-subject CV; CI, confidence interval.

## Discussion

This study was conducted to compare the bioequivalence of two products of calcium dobesilate capsules, which were assessed by conducting an open-label, randomized, single-dose, 2-period crossover bioequivalence study in healthy Chinese volunteers under fasting and fed conditions. The results of this study can provide support for the marketing of the generic domestic calcium dobesilate capsules (0.5 g/capsule) in China.

In this study, 26 and 70 healthy subjects completed the study under fasting and fed conditions respectively. A total of 75 subjects were enrolled in the fed study, of which 5 subjects dropped out before the first phase of administration (1 subject due to needle sickness and 4 due to inability to complete the high-fat, high-calorie breakfast). It suggests more attention should be paid to sample size estimates in clinical trial design under the fed conditions as the unpalatability of a high-fat, high-calorie diet in postprandial trials often results in a higher dropout rate.

During the fasting study, only 1 subject had 2 adverse reactions related to the reference preparation, with an AE incidence rate of 3.8%. The frequency of adverse events was similar between the two preparations. During the fed study, 5 adverse reactions occurred in 4 subjects related to the test preparation, with an AE rate of 5.7%, and 9 adverse reactions occurred in 9 subjects related to the reference preparation, with an AE rate of 12.9% (detailed in Table 2). There were no serious adverse events reported in all cohorts, and all AEs turned out to be cured without any treatment except one case was lost to follow-up. The results showed good safety and tolerance of single oral administration of 0.5 g calcium dobesilate capsules in healthy subjects, whether under the condition of fasting or a high-fat and high-calorie diet. Although there has been a previous study on the PK effects of dietary intake on calcium dobesilate capsules in healthy volunteers, safety data under different dietary conditions were not mentioned in the literature [12]. Our study also showed to some extent that taking the drug after a meal could not reduce the incidence of adverse reactions compared with fasting.

Comparing the PK parameters of this study with the results reported in the literatures available [11–14], the PK of various oral dosage forms (including capsules, granules, tablets, etc) of calcium dobesilate is slightly different but still comparable whether under fasting or fed conditions, especially the parameters T_max_ and t_1/2_ under fasting condition which were pretty close to the results reported in other literatures.

The values of some PK parameters changed when taking administration after taking high-fat and high-calorie meals. The T_max_ was significantly prolonged, whereas the C_max_ and AUC decreased (Table 3), which suggests food intake could delay the drug absorption of calcium dobesilate capsules and reduce its bioavailability in healthy people. The result of our study is consistent with the conclusion in the study by Liyan Liao et al [12] and reminds us the time of taking medicine should be prescribed according to treatment needs when calcium dobesilate is used clinically. However, in this study, the t_1/2_ of the fed study was longer than that of the fasting study more than 10% for reference formulation, and even more than 35% for test formulation, which may be related to the flip-flop PK properties of calcium dobesilate [24]. That is to say, the difference in the absorption process will change the terminal elimination slope of calcium dobesilate, which does not necessarily mean an inconsistent clearance rate. There are still few studies on the PK effects of dietary intake on calcium dobesilate in the human body, so further studies are needed.

In this study, under either fasting or fed conditions, the GMR of test/reference and their 90% Cis for C_max_, AUC_0-t_, and AUC_0-inf_ all fall within the range of 80.00%-125.00% (Table 4), meeting the corresponding bioequivalence criteria. To ensure that the batch factor would not affect the bioequivalence conclusion, on the basis of the bioequivalence analysis data set, the equivalence evaluation was re-performed after adding the batch factor. The results of sensitivity analysis showed that after adding batch factor, the 90% CIs of GMR for C_max_, AUC_0-t_, and AUC_0-inf_ were 88.93%-96.55%, 96.72%-101.42%, and 98.72%-104.00%, which still meet the bioequivalence criteria.

The within-subject CV (CV_W_) of primary PK parameters is considerable between fasting and fed study (Table 4). We adopted 25% as CV_W_ during study design based on previous research to estimate the sample size under fed conditions, which is higher than the result of this study, showing better control over trial factors of our study. The results of equivalence analysis under the fed condition in this study will provide a reliable reference for the sample size estimation of similar studies in the future and may help reduce research costs.

## Conclusion

In this study, the test calcium dobesilate 0.5 g capsule was bioequivalent to the reference product under both fasting and fed conditions. Taking food would slow down its rate and reduce its amount of absorption. Both formulations were generally well tolerated in this study.

## Supporting information

S1 Table The PK parameters of test preparation in the fasting study. (DOCX)

S2 Table The PK parameters of reference preparation in the fasting study. (DOCX)

S3 Table The plasma concentration (μg/mL) of calcium dobesilate in the fasting study. (DOCX)

S4 Table The PK parameters of test preparation in the fed study. (DOCX)

S5 Table The PK parameters of reference preparation in the fed study. (DOCX)

S6 Table The plasma concentration (μg/mL) of calcium dobesilate in the fed study. (DOCX)

S1 Checklist CONSORT 2010 checklist of information to include when reporting a randomised trial*. (DOC)

S1 File (DOCX)
