# Supplementary material for: A single-dose, randomized crossover study in healthy Chinese subjects to evaluate pharmacokinetics and bioequivalence of two capsules of calcium dobesilate 0.5 g under fasting and fed conditions
Source: PLoS One. 2023 Apr 21;18(4):e0284576. doi: 10.1371/journal.pone.0284576 (PMC10121042; doi:10.1371/journal.pone.0284576)
Supplement: S1 Table — (DOCX) [file pone.0284576.s001.docx]

Table S1 The PK parameters of test preparation in the fasting study

|  | | | C_max_ | AUC_0-t_ | AUC_0-∞_ | T_max_ | t_1/2z_ | λ_z_ | AUC__%Extrap_ | λ_z_first-last_ |
| --- | --- | --- | --- | --- | --- | --- | --- | --- | --- | --- |
| Subject | Sequence | Period | μg/mL | h*μg/mL | h*μg/mL | h | h | ×10^-1^1/h | % | - |
| R01 | T-R | 1 | 13.26 | 95.72 | 99.49 | 2 | 4.95 | 1.40 | 3.79 | 11-16 |
| R02 | T-R | 1 | 14.99 | 86.69 | 89.59 | 3.5 | 2.41 | 2.88 | 3.23 | 11-15 |
| R03 | R-T | 2 | 14.95 | 99.97 | 102.28 | 2 | 4.64 | 1.50 | 2.26 | 14-16 |
| R04 | R-T | 2 | 10.11 | 80.73 | 90.05 | 6 | 2.92 | 2.38 | 10.35 | 13-15 |
| R05 | R-T | 2 | 15.14 | 101.11 | 103.44 | 5 | 4.22 | 1.64 | 2.25 | 14-16 |
| R06 | T-R | 1 | 13.28 | 110.02 | 111.68 | 5 | 3.49 | 1.99 | 1.49 | 13-16 |
| R07 | R-T | 2 | 22.57 | 108.32 | 112.16 | 4 | 2.27 | 3.05 | 3.42 | 11-15 |
| R08 | T-R | 1 | 13.38 | 83.59 | 85.02 | 4 | 3.91 | 1.77 | 1.68 | 11-16 |
| R09 | T-R | 1 | 7.255 | 67.05 | 68.07 | 3.5 | 3.25 | 2.13 | 1.49 | 14-16 |
| R10 | R-T | 2 | 14.48 | 97.72 | 98.90 | 3.5 | 3.40 | 2.04 | 1.19 | 11-16 |
| R11 | T-R | 1 | 11.84 | 99.29 | 104.44 | 6 | 5.22 | 1.33 | 4.93 | 14-16 |
| R12 | R-T | 2 | 10.49 | 82.26 | 84.21 | 3.5 | 4.79 | 1.45 | 2.32 | 14-16 |
| R13 | R-T | 2 | 12.97 | 94.24 | 95.95 | 4.5 | 3.69 | 1.88 | 1.78 | 10-16 |
| R14 | T-R | 1 | 8.704 | 95.25 | 97.51 | 7 | 3.50 | 1.98 | 2.32 | 14-16 |
| R15 | R-T | 2 | 20.63 | 128.06 | 129.44 | 3.5 | 3.73 | 1.86 | 1.07 | 14-16 |
| R16 | R-T | 2 | 15.66 | 92.24 | 94.92 | 5 | 4.66 | 1.49 | 2.83 | 14-16 |
| R17 | T-R | 1 | 19.21 | 102.41 | 104.20 | 4 | 4.30 | 1.61 | 1.72 | 14-16 |
| R18 | R-T | 2 | 9.037 | 93.03 | 97.21 | 4 | 4.77 | 1.45 | 4.30 | 14-16 |
| R19 | T-R | 1 | 12.39 | 82.32 | 91.01 | 2 | 4.27 | 1.62 | 9.55 | 13-15 |
| R20 | T-R | 1 | 7.706 | 103.86 | 122.54 | 7 | 8.02 | 0.86 | 15.24 | 12-16 |
| R21 | T-R | 1 | 8.582 | 85.35 | 94.49 | 6 | 5.88 | 1.18 | 9.68 | 14-16 |
| R22 | R-T | 2 | 19.14 | 121.86 | 122.81 | 4 | 3.17 | 2.19 | 0.77 | 14-16 |
| R23 | T-R | 1 | 20.08 | 99.58 | 101.69 | 2 | 3.66 | 1.89 | 2.08 | 4-16 |
| R24 | R-T | 2 | 18.20 | 128.08 | 129.77 | 4 | 3.82 | 1.81 | 1.30 | 14-16 |
| R25 | R-T | 2 | 11.63 | 112.28 | 128.55 | 4 | 8.29 | 0.84 | 12.65 | 14-16 |
| R26 | T-R | 1 | 7.263 | 79.15 | 84.25 | 5 | 5.50 | 1.26 | 6.05 | 14-16 |
| N | | | 26 | 26 | 26 | 26 | 26 | 26 | 26 | - |
| Mean | | | 13.57 | 97.32 | 101.68 | 4.23 | 4.34 | 1.75 | 4.22 | - |
| GM | | | 12.89 | 96.22 | 100.55 | 3.99 | 4.14 | 1.67 | 2.99 | - |
| SD | | | 4.39 | 14.94 | 15.47 | 1.42 | 1.43 | 0.52 | 3.94 | - |
| CV% | | | 32.4 | 15.3 | 15.2 | 33.5 | 33.0 | 29.8 | 93.4 | - |
| Max | | | 22.57 | 128.08 | 129.77 | 7 | 8.29 | 3.05 | 15.24 | - |
| Min | | | 7.255 | 67.05 | 68.07 | 2 | 2.27 | 0.84 | 0.77 | - |
| Median | | | 13.27 | 96.72 | 99.19 | 4.00 | 4.06 | 1.71 | 2.32 | - |
| Q1 | | | 10.11 | 85.35 | 91.01 | 3.50 | 3.49 | 1.45 | 1.68 | - |
| Q3 | | | 15.66 | 103.86 | 111.68 | 5.00 | 4.79 | 1.99 | 4.93 | - |
