# Supplementary material for: A single-dose, randomized crossover study in healthy Chinese subjects to evaluate pharmacokinetics and bioequivalence of two capsules of calcium dobesilate 0.5 g under fasting and fed conditions
Source: PLoS One. 2023 Apr 21;18(4):e0284576. doi: 10.1371/journal.pone.0284576 (PMC10121042; doi:10.1371/journal.pone.0284576)
Supplement: S2 Table — (DOCX) [file pone.0284576.s002.docx]

Table S2 The PK parameters of reference preparation in the fasting study

|  | | | C_max_ | AUC_0-t_ | AUC_0-∞_ | T_max_ | t_1/2z_ | λ_z_ | AUC__%Extrap_ | λ_z_first-last_ |
| --- | --- | --- | --- | --- | --- | --- | --- | --- | --- | --- |
| Subject | Sequence | Period | μg/mL | h*μg/mL | h*μg/mL | h | h | ×10^-1^1/h | % | - |
| R01 | T-R | 2 | 10.22 | 90.76 | 96.12 | 3 | 5.35 | 1.30 | 5.57 | 14-16 |
| R02 | T-R | 2 | 13.51 | 81.98 | 90.35 | 4 | 3.68 | 1.88 | 9.27 | 11-15 |
| R03 | R-T | 1 | 9.078 | 93.24 | 100.32 | 3 | 6.11 | 1.13 | 7.06 | 14-16 |
| R04 | R-T | 1 | 10.13 | 99.37 | 100.29 | 6 | 2.95 | 2.35 | 0.92 | 13-16 |
| R05 | R-T | 1 | 10.99 | 92.65 | 97.21 | 6 | 4.74 | 1.46 | 4.70 | 14-16 |
| R06 | T-R | 2 | 19.90 | 120.68 | 121.85 | 4 | 3.50 | 1.98 | 0.96 | 14-16 |
| R07 | R-T | 1 | 15.16 | 116.59 | 120.82 | 5 | 4.95 | 1.40 | 3.50 | 14-16 |
| R08 | T-R | 2 | 9.858 | 85.37 | 91.47 | 4.5 | 6.29 | 1.10 | 6.67 | 14-16 |
| R09 | T-R | 2 | 14.74 | 75.11 | 78.74 | 3 | 2.94 | 2.36 | 4.61 | 7-15 |
| R10 | R-T | 1 | 7.879 | 73.62 | 74.50 | 5.5 | 2.99 | 2.32 | 1.18 | 14-16 |
| R11 | T-R | 2 | 16.83 | 108.66 | 110.35 | 5 | 3.89 | 1.78 | 1.53 | 14-16 |
| R12 | R-T | 1 | 10.23 | 75.92 | 82.33 | 2 | 7.38 | 0.94 | 7.78 | 14-16 |
| R13 | R-T | 1 | 10.04 | 81.07 | 85.26 | 5.5 | 5.11 | 1.36 | 4.92 | 14-16 |
| R14 | T-R | 2 | 10.62 | 109.66 | 113.32 | 6 | 3.92 | 1.77 | 3.23 | 14-16 |
| R15 | R-T | 1 | 14.38 | 111.76 | 117.06 | 5 | 5.14 | 1.35 | 4.53 | 14-16 |
| R16 | R-T | 1 | 13.80 | 81.41 | 85.58 | 4 | 5.69 | 1.22 | 4.87 | 14-16 |
| R17 | T-R | 2 | 19.57 | 106.89 | 108.08 | 4 | 3.58 | 1.93 | 1.10 | 14-16 |
| R18 | R-T | 1 | 7.595 | 91.15 | 97.03 | 4 | 5.19 | 1.34 | 6.06 | 13-16 |
| R19 | T-R | 2 | 15.49 | 96.65 | 101.66 | 3 | 2.91 | 2.38 | 4.93 | 9-15 |
| R20 | T-R | 2 | 10.27 | 104.54 | 111.48 | 6 | 5.51 | 1.26 | 6.23 | 14-16 |
| R21 | T-R | 2 | 6.718 | 78.61 | 92.27 | 6 | 7.02 | 0.99 | 14.80 | 14-16 |
| R22 | R-T | 1 | 12.99 | 112.66 | 114.45 | 6 | 3.62 | 1.91 | 1.57 | 14-16 |
| R23 | T-R | 2 | 17.86 | 103.65 | 108.96 | 3.5 | 6.83 | 1.01 | 4.87 | 14-16 |
| R24 | R-T | 1 | 18.52 | 126.66 | 129.00 | 4 | 4.09 | 1.69 | 1.82 | 14-16 |
| R25 | R-T | 1 | 10.85 | 119.59 | 126.45 | 4 | 5.04 | 1.37 | 5.42 | 11-16 |
| R26 | T-R | 2 | 10.01 | 82.98 | 87.62 | 5.5 | 5.76 | 1.20 | 5.29 | 14-16 |
| N | | | 26 | 26 | 26 | 26 | 26 | 26 | 26 | - |
| Mean | | | 12.59 | 96.97 | 101.64 | 4.52 | 4.78 | 1.57 | 4.75 | - |
| GM | | | 12.06 | 95.73 | 100.55 | 4.35 | 4.60 | 1.51 | 3.77 | - |
| SD | | | 3.77 | 15.83 | 15.10 | 1.19 | 1.33 | 0.45 | 3.05 | - |
| CV% | | | 29.9 | 16.3 | 14.9 | 26.3 | 27.9 | 29.0 | 64.3 | - |
| Max | | | 19.90 | 126.66 | 129.00 | 6 | 7.38 | 2.38 | 14.80 | - |
| Min | | | 6.718 | 73.62 | 74.50 | 2 | 2.91 | 0.94 | 0.92 | - |
| Median | | | 10.92 | 94.95 | 100.31 | 4.25 | 4.99 | 1.39 | 4.87 | - |
| Q1 | | | 10.04 | 81.98 | 90.35 | 4.00 | 3.62 | 1.22 | 1.82 | - |
| Q3 | | | 15.16 | 109.66 | 113.32 | 5.50 | 5.69 | 1.91 | 6.06 | - |
