# Supplementary material for: A single-dose, randomized crossover study in healthy Chinese subjects to evaluate pharmacokinetics and bioequivalence of two capsules of calcium dobesilate 0.5 g under fasting and fed conditions
Source: PLoS One. 2023 Apr 21;18(4):e0284576. doi: 10.1371/journal.pone.0284576 (PMC10121042; doi:10.1371/journal.pone.0284576)
Supplement: S3 Table — (DOCX) [file pone.0284576.s003.docx]

Table S3 The plasma concentration (μg/mL) of calcium dobesilate in the fasting study

|  | T | | | | | | | | | R | | | | | | | | |
| --- | --- | --- | --- | --- | --- | --- | --- | --- | --- | --- | --- | --- | --- | --- | --- | --- | --- | --- |
| Time(h) | N | N_BQL_ | Mean | SD | CV% | Median | Min | Max |  | N | N_BQL_ | Mean | SD | CV% | Median | Min | Max |  |
| 0 | 26 | 26 | 0 | 0 | NA | 0 | 0 | 0 |  | 26 | 26 | 0 | 0 | NA | 0 | 0 | 0 |  |
| 1 | 26 | 0 | 4.34 | 2.22 | 51.1 | 3.87 | 1.650 | 11.06 |  | 26 | 0 | 3.41 | 1.59 | 46.7 | 3.11 | 1.945 | 9.881 |  |
| 2 | 26 | 0 | 7.51 | 4.10 | 54.6 | 6.44 | 2.672 | 20.08 |  | 26 | 0 | 6.22 | 3.61 | 58.1 | 4.75 | 2.395 | 15.43 |  |
| 3 | 26 | 0 | 9.45 | 4.07 | 43.1 | 9.86 | 2.131 | 17.74 |  | 26 | 0 | 8.89 | 4.33 | 48.7 | 7.90 | 2.346 | 19.22 |  |
| 3.5 | 26 | 0 | 10.74 | 4.64 | 43.2 | 10.94 | 2.175 | 20.63 |  | 26 | 0 | 9.71 | 4.40 | 45.3 | 9.23 | 2.337 | 18.44 |  |
| 4 | 26 | 0 | 11.38 | 5.12 | 44.9 | 10.75 | 2.528 | 22.57 |  | 26 | 0 | 10.68 | 4.82 | 45.2 | 10.06 | 2.375 | 19.90 |  |
| 4.5 | 26 | 0 | 11.10 | 4.46 | 40.2 | 10.77 | 3.766 | 18.77 |  | 26 | 0 | 10.19 | 3.88 | 38.0 | 9.67 | 2.799 | 17.00 |  |
| 5 | 26 | 0 | 10.94 | 4.16 | 38.0 | 9.76 | 4.731 | 18.82 |  | 26 | 0 | 10.14 | 3.47 | 34.2 | 9.28 | 3.759 | 16.83 |  |
| 5.5 | 26 | 0 | 10.50 | 3.59 | 34.2 | 9.94 | 3.796 | 17.61 |  | 26 | 0 | 10.18 | 2.90 | 28.4 | 9.49 | 5.785 | 15.84 |  |
| 6 | 26 | 0 | 9.58 | 2.69 | 28.1 | 9.20 | 4.015 | 14.92 |  | 26 | 0 | 9.55 | 2.64 | 27.7 | 9.04 | 5.330 | 14.74 |  |
| 7 | 26 | 0 | 7.61 | 1.83 | 24.0 | 7.56 | 3.780 | 10.28 |  | 26 | 0 | 7.89 | 2.14 | 27.2 | 7.72 | 4.295 | 12.01 |  |
| 8 | 26 | 0 | 6.19 | 1.40 | 22.5 | 6.45 | 3.582 | 8.273 |  | 26 | 0 | 6.52 | 1.70 | 26.0 | 6.40 | 3.721 | 9.748 |  |
| 10 | 26 | 0 | 4.24 | 1.13 | 26.5 | 4.38 | 2.382 | 6.697 |  | 26 | 0 | 4.51 | 1.27 | 28.2 | 4.44 | 2.055 | 6.751 |  |
| 12 | 26 | 0 | 2.84 | 0.94 | 33.1 | 2.70 | 1.444 | 4.934 |  | 26 | 0 | 3.09 | 0.95 | 30.7 | 3.11 | 1.408 | 5.321 |  |
| 14 | 26 | 0 | 1.95 | 0.82 | 42.0 | 1.78 | 0.8328 | 4.262 |  | 26 | 0 | 2.22 | 0.80 | 36.0 | 2.10 | 0.8551 | 3.926 |  |
| 24 | 26 | 4 | 0.43 | 0.39 | 91.3 | 0.33 | 0 | 1.614 |  | 26 | 3 | 0.52 | 0.32 | 61.8 | 0.56 | 0 | 1.348 |  |
