# Supplementary material for: A single-dose, randomized crossover study in healthy Chinese subjects to evaluate pharmacokinetics and bioequivalence of two capsules of calcium dobesilate 0.5 g under fasting and fed conditions
Source: PLoS One. 2023 Apr 21;18(4):e0284576. doi: 10.1371/journal.pone.0284576 (PMC10121042; doi:10.1371/journal.pone.0284576)
Supplement: S4 Table — (DOCX) [file pone.0284576.s004.docx]

Table S4 The PK parameters of test preparation in the fed study

|  | | | C_max_ | AUC_0-t_ | AUC_0-∞_ | T_max_ | t_1/2z_ | λ_z_ | AUC__%Extrap_ | λ_z_first-last_ |
| --- | --- | --- | --- | --- | --- | --- | --- | --- | --- | --- |
| Subject | Sequence | Period | μg/mL | h*μg/mL | h*μg/mL | h | h | ×10^-1^1/h | % | - |
| R54 | T-R | 1 | 6.453 | 82.50 | 89.05 | 5.5 | 5.20 | 1.33 | 7.35 | 14-16 |
| R55 | R-T | 2 | 4.746 | 62.80 | 87.97 | 6 | 11.66 | 0.59 | 28.61 | 14-16 |
| R56 | R-T | 2 | 5.575 | 81.15 | 95.42 | 10 | 6.87 | 1.01 | 14.96 | 14-16 |
| R57 | R-T | 2 | 8.002 | 93.99 | 104.14 | 4 | 6.42 | 1.08 | 9.75 | 12-16 |
| R58 | T-R | 1 | 6.658 | 74.13 | 79.09 | 5.5 | 5.24 | 1.32 | 6.26 | 14-16 |
| R59 | R-T | 2 | 7.632 | 97.45 | 126.29 | 5 | 9.63 | 0.72 | 22.83 | 9-16 |
| R60 | T-R | 1 | 6.392 | 85.86 | 97.39 | 6 | 6.49 | 1.07 | 11.84 | 13-16 |
| R61 | R-T | 2 | 4.334 | 50.88 | 60.26 | 4 | 8.40 | 0.83 | 15.56 | 13-16 |
| R62 | R-T | 2 | 7.618 | 71.10 | 73.74 | 4.5 | 4.36 | 1.59 | 3.58 | 9-16 |
| R63 | T-R | 1 | 8.164 | 76.99 | 82.66 | 3 | 6.14 | 1.13 | 6.86 | 14-16 |
| R64 | T-R | 1 | 6.706 | 71.52 | 77.11 | 10 | 4.80 | 1.44 | 7.24 | 14-16 |
| R65 | T-R | 1 | 11.19 | 85.19 | 87.51 | 4.5 | 4.08 | 1.70 | 2.64 | 8-16 |
| R66 | R-T | 2 | 8.184 | 99.26 | 102.62 | 5 | 3.98 | 1.74 | 3.28 | 14-16 |
| R67 | T-R | 1 | 4.439 | 55.48 | 62.09 | 3.5 | 6.81 | 1.02 | 10.64 | 9-16 |
| R68 | R-T | 2 | 7.193 | 67.30 | 68.55 | 3 | 3.62 | 1.92 | 1.82 | 12-16 |
| R69 | T-R | 1 | 6.593 | 83.10 | 98.68 | 6 | 7.24 | 0.96 | 15.78 | 14-16 |
| R70 | R-T | 2 | 4.064 | 58.60 | - | 14 | - | - | - | - |
| R71 | R-T | 2 | 13.02 | 108.54 | 113.28 | 5 | 4.52 | 1.53 | 4.19 | 9-16 |
| R72 | T-R | 1 | 4.551 | 60.72 | 73.99 | 10 | 7.11 | 0.97 | 17.93 | 14-16 |
| R73 | T-R | 1 | 5.241 | 69.09 | 75.46 | 5.5 | 5.50 | 1.26 | 8.43 | 14-16 |
| R74 | R-T | 2 | 5.156 | 69.73 | 77.75 | 7 | 5.61 | 1.24 | 10.31 | 14-16 |
| R75 | R-T | 2 | 5.946 | 88.49 | 109.06 | 7 | 7.78 | 0.89 | 18.86 | 14-16 |
| R76 | T-R | 1 | 5.518 | 66.56 | 72.26 | 8 | 5.25 | 1.32 | 7.90 | 13-16 |
| R77 | T-R | 1 | 6.799 | 76.11 | 83.18 | 5.5 | 6.26 | 1.11 | 8.50 | 14-16 |
| R78 | T-R | 1 | 6.962 | 81.60 | 91.14 | 7 | 5.83 | 1.19 | 10.47 | 14-16 |
| R79 | R-T | 2 | 5.338 | 80.31 | 87.96 | 10 | 5.08 | 1.36 | 8.69 | 14-16 |
| R80 | R-T | 2 | 6.403 | 80.10 | 85.70 | 8 | 4.92 | 1.41 | 6.54 | 14-16 |
| R81 | T-R | 1 | 8.323 | 93.50 | 99.02 | 5 | 5.46 | 1.27 | 5.58 | 14-16 |
| R82 | R-T | 2 | 6.706 | 93.75 | 113.19 | 7 | 7.85 | 0.88 | 17.17 | 14-16 |
| R83 | T-R | 1 | 7.207 | 77.40 | 84.08 | 5 | 5.93 | 1.17 | 7.94 | 10-16 |
| R84 | R-T | 2 | 6.602 | 81.41 | 91.18 | 4 | 6.64 | 1.04 | 10.72 | 14-16 |
| R85 | T-R | 1 | 5.018 | 72.93 | 98.50 | 6 | 11.26 | 0.62 | 25.96 | 14-16 |
| R86 | T-R | 1 | 7.860 | 92.50 | 100.14 | 6 | 5.53 | 1.25 | 7.63 | 14-16 |
| R87 | R-T | 2 | 6.741 | 64.23 | 71.86 | 5 | 6.91 | 1.00 | 10.62 | 13-16 |
| R88 | R-T | 2 | 5.204 | 65.73 | 72.03 | 4.5 | 5.80 | 1.19 | 8.74 | 14-16 |
| R89 | R-T | 2 | 5.741 | 87.05 | 98.32 | 10 | 6.12 | 1.13 | 11.46 | 14-16 |
| R90 | T-R | 1 | 5.068 | 61.86 | 71.39 | 10 | 6.62 | 1.05 | 13.36 | 14-16 |
| R91 | R-T | 2 | 6.784 | 72.36 | 74.29 | 5 | 3.67 | 1.89 | 2.61 | 13-16 |
| R92 | T-R | 1 | 7.527 | 78.54 | 89.92 | 3 | 7.29 | 0.95 | 12.66 | 11-16 |
| R93 | R-T | 2 | 8.426 | 104.38 | 117.95 | 4.5 | 7.10 | 0.98 | 11.51 | 12-16 |
| R94 | T-R | 1 | 6.428 | 93.59 | 107.08 | 6 | 6.76 | 1.03 | 12.60 | 13-16 |
| R96 | R-T | 2 | 4.637 | 68.85 | 80.73 | 5.5 | 6.88 | 1.01 | 14.72 | 14-16 |
| R99 | T-R | 1 | 6.041 | 76.34 | 88.62 | 4.5 | 7.39 | 0.94 | 13.86 | 13-16 |
| R100 | R-T | 2 | 11.12 | 86.69 | 94.11 | 4.5 | 5.85 | 1.19 | 7.89 | 11-16 |
| R101 | R-T | 2 | 8.913 | 88.47 | 91.81 | 4.5 | 4.41 | 1.57 | 3.64 | 10-16 |
| R103 | T-R | 1 | 7.069 | 100.71 | 113.63 | 6 | 6.36 | 1.09 | 11.38 | 14-16 |
| R104 | T-R | 1 | 6.751 | 85.67 | 95.29 | 4.5 | 6.41 | 1.08 | 10.10 | 14-16 |
| R105 | T-R | 1 | 9.476 | 106.52 | 115.21 | 8 | 5.09 | 1.36 | 7.54 | 13-16 |
| R106 | T-R | 1 | 8.911 | 77.25 | 78.89 | 4.5 | 3.73 | 1.86 | 2.08 | 9-16 |
| R107 | R-T | 2 | 4.858 | 64.32 | 73.00 | 4 | 6.72 | 1.03 | 11.88 | 12-16 |
| R108 | R-T | 2 | 7.673 | 82.14 | 83.58 | 4.5 | 3.33 | 2.08 | 1.73 | 14-16 |
| R109 | T-R | 1 | 9.707 | 81.22 | 87.62 | 3.5 | 6.53 | 1.06 | 7.31 | 13-16 |
| R110 | T-R | 1 | 5.389 | 79.55 | 91.48 | 4.5 | 6.53 | 1.06 | 13.04 | 14-16 |
| R111 | R-T | 2 | 5.160 | 75.24 | 104.57 | 8 | 10.63 | 0.65 | 28.06 | 14-16 |
| R112 | R-T | 2 | 5.212 | 74.66 | 79.88 | 6 | 4.50 | 1.54 | 6.53 | 14-16 |
| R113 | R-T | 2 | 7.210 | 75.61 | 81.94 | 3.5 | 6.02 | 1.15 | 7.72 | 14-16 |
| R114 | T-R | 1 | 6.737 | 98.91 | 126.82 | 10 | 8.83 | 0.78 | 22.00 | 14-16 |
| R115 | T-R | 1 | 4.925 | 85.06 | 102.33 | 8 | 7.45 | 0.93 | 16.87 | 14-16 |
| R116 | R-T | 2 | 5.725 | 76.83 | 84.37 | 8 | 5.51 | 1.26 | 8.93 | 13-16 |
| R117 | R-T | 2 | 7.755 | 81.61 | 85.56 | 5.5 | 4.60 | 1.51 | 4.61 | 11-16 |
| R118 | R-T | 2 | 9.460 | 105.20 | 131.57 | 6 | 9.86 | 0.70 | 20.04 | 14-16 |
| R119 | T-R | 1 | 7.285 | 79.22 | 89.28 | 6 | 6.30 | 1.10 | 11.27 | 11-16 |
| R120 | T-R | 1 | 5.594 | 71.47 | 73.67 | 4.5 | 3.74 | 1.85 | 2.98 | 14-16 |
| R121 | T-R | 1 | 4.584 | 66.27 | 80.60 | 7 | 7.78 | 0.89 | 17.77 | 14-16 |
| R122 | R-T | 2 | 5.959 | 85.17 | 102.02 | 7 | 8.01 | 0.87 | 16.52 | 13-16 |
| R123 | T-R | 1 | 6.693 | 83.42 | 96.22 | 6 | 6.72 | 1.03 | 13.31 | 13-16 |
| R124 | R-T | 2 | 6.181 | 84.48 | 89.97 | 6 | 4.85 | 1.43 | 6.10 | 14-16 |
| T53 | T-R | 1 | 4.999 | 69.80 | 80.34 | 7 | 6.43 | 1.08 | 13.12 | 14-16 |
| T95 | T-R | 1 | 7.646 | 90.71 | 99.35 | 8 | 5.53 | 1.25 | 8.69 | 13-16 |
| T98 | T-R | 1 | 5.180 | 62.39 | 66.82 | 4.5 | 5.27 | 1.32 | 6.64 | 12-16 |
| N(NMiss) | | | 70(0) | 70(0) | 63(7) * | 70(0) | 63(7) * | 63(7) * | 63(7) * | - |
| Mean | | | 6.71 | 79.74 | 88.01 | 6.04 | 5.89 | 1.23 | 9.50 | - |
| GM | | | 6.51 | 78.74 | 86.98 | 5.72 | 5.76 | 1.20 | 8.25 | - |
| SD | | | 1.74 | 12.67 | 13.61 | 2.12 | 1.22 | 0.29 | 4.45 | - |
| CV% | | | 25.9 | 15.9 | 15.5 | 35.1 | 20.8 | 23.7 | 46.8 | - |
| Max | | | 13.02 | 108.54 | 117.95 | 14 | 8.40 | 2.08 | 18.86 | - |
| Min | | | 4.064 | 50.88 | 60.26 | 3 | 3.33 | 0.83 | 1.73 | - |
| Median | | | 6.63 | 79.82 | 87.62 | 5.50 | 6.02 | 1.15 | 8.74 | - |
| Q1 | | | 5.24 | 71.10 | 77.75 | 4.50 | 5.08 | 1.03 | 6.54 | - |
| Q3 | | | 7.63 | 86.69 | 98.32 | 7.00 | 6.76 | 1.36 | 12.66 | - |
