# Supplementary material for: A single-dose, randomized crossover study in healthy Chinese subjects to evaluate pharmacokinetics and bioequivalence of two capsules of calcium dobesilate 0.5 g under fasting and fed conditions
Source: PLoS One. 2023 Apr 21;18(4):e0284576. doi: 10.1371/journal.pone.0284576 (PMC10121042; doi:10.1371/journal.pone.0284576)
Supplement: S5 Table — (DOCX) [file pone.0284576.s005.docx]

Table S5 The PK parameters of reference preparation in the fed study

|  | | | C_max_ | AUC_0-t_ | AUC_0-∞_ | T_max_ | t_1/2z_ | λ_z_ | AUC__%Extrap_ | λ_z_first-last_ |
| --- | --- | --- | --- | --- | --- | --- | --- | --- | --- | --- |
| Subject | Sequence | Period | μg/mL | h*μg/mL | h*μg/mL | h | h | ×10^-1^1/h | % | - |
| R54 | T-R | 2 | 6.168 | 77.38 | 91.19 | 7 | 7.53 | 0.92 | 15.14 | 14-16 |
| R55 | R-T | 1 | 7.829 | 76.10 | 86.42 | 4.5 | 7.74 | 0.90 | 11.94 | 14-16 |
| R56 | R-T | 1 | 7.201 | 79.21 | 93.28 | 6 | 7.08 | 0.98 | 15.08 | 11-16 |
| R57 | R-T | 1 | 7.278 | 92.90 | 101.38 | 4.5 | 5.87 | 1.18 | 8.37 | 14-16 |
| R58 | T-R | 2 | 5.937 | 75.33 | 78.93 | 8 | 4.40 | 1.57 | 4.56 | 13-16 |
| R59 | R-T | 1 | 7.488 | 89.00 | 126.01 | 5.5 | 14.69 | 0.47 | 29.37 | 14-16 |
| R60 | T-R | 2 | 5.887 | 63.05 | 64.65 | 4 | 3.77 | 1.84 | 2.48 | 14-16 |
| R61 | R-T | 1 | 6.229 | 54.49 | 57.61 | 4 | 5.35 | 1.30 | 5.40 | 7-16 |
| R62 | R-T | 1 | 7.017 | 86.27 | 92.87 | 5.5 | 5.36 | 1.29 | 7.10 | 14-16 |
| R63 | T-R | 2 | 8.701 | 71.41 | 73.27 | 6 | 3.79 | 1.83 | 2.54 | 11-16 |
| R64 | T-R | 2 | 8.339 | 83.76 | 85.15 | 6 | 3.17 | 2.18 | 1.63 | 14-16 |
| R65 | T-R | 2 | 10.00 | 87.95 | 89.58 | 4.5 | 3.62 | 1.92 | 1.82 | 14-16 |
| R66 | R-T | 1 | 8.235 | 88.56 | 94.68 | 6 | 4.66 | 1.49 | 6.47 | 14-16 |
| R67 | T-R | 2 | 5.855 | 63.69 | 66.75 | 3.5 | 4.72 | 1.47 | 4.58 | 13-16 |
| R68 | R-T | 1 | 6.743 | 69.69 | 71.37 | 4.5 | 3.82 | 1.82 | 2.35 | 11-16 |
| R69 | T-R | 2 | 6.171 | 72.05 | 73.13 | 5 | 2.98 | 2.33 | 1.48 | 14-16 |
| R70 | R-T | 1 | 5.570 | 81.00 | 94.85 | 5 | 6.82 | 1.02 | 14.61 | 14-16 |
| R71 | R-T | 1 | 12.67 | 113.58 | 116.08 | 5 | 3.87 | 1.79 | 2.16 | 14-16 |
| R72 | T-R | 2 | 6.151 | 68.03 | 74.19 | 3.5 | 6.00 | 1.15 | 8.31 | 12-16 |
| R73 | T-R | 2 | 6.560 | 75.02 | 78.15 | 3.5 | 4.32 | 1.60 | 4.01 | 13-16 |
| R74 | R-T | 1 | 5.583 | 76.37 | 82.71 | 10 | 5.18 | 1.34 | 7.67 | 14-16 |
| R75 | R-T | 1 | 6.202 | 79.75 | 98.28 | 8 | 7.71 | 0.90 | 18.85 | 13-16 |
| R76 | T-R | 2 | 5.615 | 47.48 | 51.13 | 4 | 6.05 | 1.15 | 7.12 | 11-16 |
| R77 | T-R | 2 | 7.308 | 81.28 | 87.97 | 6 | 5.51 | 1.26 | 7.61 | 14-16 |
| R78 | T-R | 2 | 6.247 | 80.66 | 89.89 | 4 | 6.09 | 1.14 | 10.27 | 14-16 |
| R79 | R-T | 1 | 4.859 | 69.99 | - | 14 | - | - | - | - |
| R80 | R-T | 1 | 8.031 | 81.10 | 83.40 | 8 | 3.78 | 1.83 | 2.75 | 14-16 |
| R81 | T-R | 2 | 7.737 | 96.64 | 99.59 | 8 | 4.05 | 1.71 | 2.96 | 13-16 |
| R82 | R-T | 1 | 5.554 | 84.36 | 97.99 | 7 | 6.51 | 1.06 | 13.91 | 14-16 |
| R83 | T-R | 2 | 6.790 | 81.25 | 89.82 | 3 | 6.49 | 1.07 | 9.55 | 9-16 |
| R84 | R-T | 1 | 6.733 | 80.91 | 92.76 | 5 | 7.36 | 0.94 | 12.78 | 11-16 |
| R85 | T-R | 2 | 4.921 | 76.81 | 88.74 | 3 | 7.03 | 0.99 | 13.44 | 14-16 |
| R86 | T-R | 2 | 9.481 | 82.66 | 83.78 | 3 | 3.57 | 1.94 | 1.33 | 14-16 |
| R87 | R-T | 1 | 8.626 | 72.74 | 77.58 | 4 | 5.32 | 1.30 | 6.24 | 7-16 |
| R88 | R-T | 1 | 6.553 | 67.79 | 73.29 | 3.5 | 6.20 | 1.12 | 7.50 | 14-16 |
| R89 | R-T | 1 | 6.183 | 82.33 | 90.87 | 10 | 5.32 | 1.30 | 9.40 | 14-16 |
| R90 | T-R | 2 | 5.002 | 61.61 | 66.87 | 7 | 5.57 | 1.24 | 7.86 | 14-16 |
| R91 | R-T | 1 | 5.177 | 77.76 | 89.54 | 10 | 6.12 | 1.13 | 13.15 | 14-16 |
| R92 | T-R | 2 | 7.160 | 77.38 | 79.92 | 3.5 | 4.20 | 1.65 | 3.18 | 13-16 |
| R93 | R-T | 1 | 6.097 | 91.83 | 113.05 | 5.5 | 8.34 | 0.83 | 18.78 | 14-16 |
| R94 | T-R | 2 | 8.378 | 97.03 | 101.22 | 6 | 4.46 | 1.56 | 4.14 | 14-16 |
| R96 | R-T | 1 | 6.454 | 72.20 | 78.32 | 4 | 5.92 | 1.17 | 7.82 | 11-16 |
| R99 | T-R | 2 | 5.560 | 73.14 | 81.46 | 10 | 6.08 | 1.14 | 10.22 | 14-16 |
| R100 | R-T | 1 | 10.98 | 92.02 | 95.98 | 4 | 4.67 | 1.48 | 4.12 | 7-16 |
| R101 | R-T | 1 | 6.673 | 81.26 | 86.20 | 3.5 | 4.82 | 1.44 | 5.72 | 13-16 |
| R103 | T-R | 2 | 14.29 | 130.28 | 134.75 | 4 | 4.40 | 1.58 | 3.31 | 13-16 |
| R104 | T-R | 2 | 7.627 | 92.09 | 97.84 | 6 | 5.22 | 1.33 | 5.87 | 12-16 |
| R105 | T-R | 2 | 12.09 | 116.98 | 120.70 | 5 | 4.30 | 1.61 | 3.08 | 14-16 |
| R106 | T-R | 2 | 9.496 | 65.83 | 69.06 | 4 | 2.16 | 3.21 | 4.68 | 13-15 |
| R107 | R-T | 1 | 4.951 | 66.59 | 71.81 | 6 | 5.36 | 1.29 | 7.28 | 14-16 |
| R108 | R-T | 1 | 6.475 | 78.14 | 79.61 | 7 | 3.38 | 2.05 | 1.84 | 13-16 |
| R109 | T-R | 2 | 9.821 | 87.45 | 88.83 | 6 | 3.18 | 2.18 | 1.55 | 13-16 |
| R110 | T-R | 2 | 6.023 | 82.48 | 98.63 | 6 | 7.36 | 0.94 | 16.38 | 14-16 |
| R111 | R-T | 1 | 7.633 | 86.44 | 90.18 | 4.5 | 4.44 | 1.56 | 4.14 | 14-16 |
| R112 | R-T | 1 | 6.971 | 80.97 | 95.31 | 4 | 8.45 | 0.82 | 15.05 | 12-16 |
| R113 | R-T | 1 | 4.394 | 64.71 | 96.25 | 5.5 | 13.25 | 0.52 | 32.78 | 13-16 |
| R114 | T-R | 2 | 9.936 | 105.22 | 106.66 | 5 | 3.24 | 2.14 | 1.35 | 14-16 |
| R115 | T-R | 2 | 8.954 | 86.99 | 88.38 | 5 | 3.51 | 1.97 | 1.57 | 11-16 |
| R116 | R-T | 1 | 7.424 | 82.83 | 89.00 | 5.5 | 5.41 | 1.28 | 6.94 | 10-16 |
| R117 | R-T | 1 | 7.766 | 67.54 | 69.29 | 5.5 | 4.40 | 1.58 | 2.52 | 14-16 |
| R118 | R-T | 1 | 7.420 | 102.08 | 123.03 | 5 | 7.88 | 0.88 | 17.02 | 14-16 |
| R119 | T-R | 2 | 7.492 | 91.48 | 94.80 | 8 | 4.11 | 1.68 | 3.50 | 13-16 |
| R120 | T-R | 2 | 6.110 | 59.79 | 74.35 | 5.5 | 4.43 | 1.56 | 19.58 | 12-15 |
| R121 | T-R | 2 | 5.741 | 70.46 | 79.01 | 5 | 6.67 | 1.04 | 10.83 | 14-16 |
| R122 | R-T | 1 | 7.447 | 85.76 | 101.09 | 4 | 7.52 | 0.92 | 15.17 | 7-16 |
| R123 | T-R | 2 | 8.060 | 87.63 | 92.25 | 4.5 | 5.19 | 1.34 | 5.00 | 13-16 |
| R124 | R-T | 1 | 7.322 | 73.75 | 77.62 | 3.5 | 5.10 | 1.36 | 4.98 | 14-16 |
| T53 | T-R | 2 | 4.460 | 67.45 | 80.01 | 6 | 7.28 | 0.95 | 15.70 | 14-16 |
| T95 | T-R | 2 | 9.592 | 100.56 | 105.82 | 5.5 | 4.93 | 1.41 | 4.97 | 14-16 |
| T98 | T-R | 2 | 6.118 | 81.74 | 91.37 | 4.5 | 6.36 | 1.09 | 10.54 | 12-16 |
| N(NMiss) | | | 70(0) | 70(0) | 67(3) * | 70(0) | 67(3) * | 67(3) * | 67(3) * | - |
| Mean | | | 7.25 | 80.71 | 87.81 | 5.51 | 5.31 | 1.42 | 7.60 | - |
| GM | | | 7.04 | 79.58 | 86.55 | 5.22 | 5.10 | 1.36 | 5.86 | - |
| SD | | | 1.89 | 13.92 | 15.04 | 2.00 | 1.48 | 0.44 | 5.15 | - |
| CV% | | | 26.1 | 17.2 | 17.1 | 36.3 | 27.8 | 30.9 | 67.8 | - |
| Max | | | 14.29 | 130.28 | 134.75 | 14 | 8.45 | 3.21 | 19.58 | - |
| Min | | | 4.394 | 47.48 | 51.13 | 3 | 2.16 | 0.82 | 1.33 | - |
| Median | | | 6.88 | 80.94 | 88.83 | 5.00 | 5.22 | 1.33 | 6.47 | - |
| Q1 | | | 6.10 | 72.05 | 78.15 | 4.00 | 4.20 | 1.09 | 3.18 | - |
| Q3 | | | 8.03 | 87.45 | 95.31 | 6.00 | 6.36 | 1.65 | 10.83 | - |
