# Supplementary material for: A single-dose, randomized crossover study in healthy Chinese subjects to evaluate pharmacokinetics and bioequivalence of two capsules of calcium dobesilate 0.5 g under fasting and fed conditions
Source: PLoS One. 2023 Apr 21;18(4):e0284576. doi: 10.1371/journal.pone.0284576 (PMC10121042; doi:10.1371/journal.pone.0284576)
Supplement: S6 Table — (DOCX) [file pone.0284576.s006.docx]

Table S6 The plasma concentration (μg/mL) of calcium dobesilate in the fed study

|  | T | | | | | | | | | R | | | | | | | | |
| --- | --- | --- | --- | --- | --- | --- | --- | --- | --- | --- | --- | --- | --- | --- | --- | --- | --- | --- |
| Time (h) | N | N_BQL_ | Mean | SD | CV% | Median | Min | Max |  | N | N_BQL_ | Mean | SD | CV% | Median | Min | Max |  |
| 0 | 70 | 70 | 0 | 0 | NA | 0 | 0 | 0 |  | 70 | 70 | 0 | 0 | NA | 0 | 0 | 0 |  |
| 1 | 70 | 15 | 1.09 | 1.01 | 93.1 | 0.93 | 0 | 3.919 |  | 70 | 9 | 1.47 | 1.08 | 73.3 | 1.37 | 0 | 3.998 |  |
| 2 | 70 | 2 | 2.51 | 1.71 | 68.2 | 2.23 | 0 | 7.510 |  | 70 | 3 | 3.51 | 1.85 | 52.6 | 3.43 | 0 | 8.914 |  |
| 3 | 70 | 0 | 4.17 | 2.19 | 52.5 | 3.92 | 0.2495 | 9.114 |  | 70 | 0 | 5.35 | 2.20 | 41.2 | 5.46 | 0.7724 | 12.28 |  |
| 3.5 | 70 | 0 | 4.85 | 2.15 | 44.4 | 4.72 | 0.3328 | 11.21 |  | 70 | 0 | 5.87 | 2.20 | 37.5 | 6.14 | 1.367 | 13.06 |  |
| 4 | 70 | 0 | 5.43 | 2.15 | 39.6 | 5.18 | 0.3878 | 12.07 |  | 70 | 0 | 6.32 | 2.17 | 34.3 | 6.20 | 1.889 | 14.29 |  |
| 4.5 | 70 | 0 | 5.74 | 2.09 | 36.4 | 5.46 | 0.5098 | 12.22 |  | 70 | 0 | 6.36 | 2.05 | 32.2 | 6.06 | 2.409 | 13.20 |  |
| 5 | 70 | 0 | 5.84 | 1.98 | 33.9 | 5.59 | 0.6569 | 13.02 |  | 70 | 0 | 6.45 | 1.91 | 29.7 | 6.22 | 2.685 | 12.67 |  |
| 5.5 | 70 | 0 | 5.92 | 1.76 | 29.7 | 5.72 | 0.8038 | 12.12 |  | 70 | 0 | 6.46 | 1.90 | 29.5 | 6.24 | 3.080 | 12.32 |  |
| 6 | 70 | 0 | 5.91 | 1.61 | 27.2 | 5.82 | 0.8550 | 11.05 |  | 70 | 0 | 6.40 | 1.82 | 28.5 | 6.07 | 3.007 | 12.32 |  |
| 7 | 70 | 0 | 5.66 | 1.30 | 22.9 | 5.58 | 1.031 | 8.362 |  | 70 | 0 | 6.10 | 1.61 | 26.4 | 5.87 | 2.867 | 10.64 |  |
| 8 | 70 | 0 | 5.42 | 1.22 | 22.5 | 5.38 | 1.182 | 9.476 |  | 70 | 0 | 5.64 | 1.39 | 24.7 | 5.54 | 2.566 | 9.346 |  |
| 10 | 70 | 0 | 4.81 | 1.02 | 21.3 | 4.93 | 2.277 | 7.999 |  | 70 | 0 | 4.73 | 1.10 | 23.1 | 4.53 | 2.045 | 7.484 |  |
| 12 | 70 | 0 | 3.93 | 0.86 | 21.8 | 3.95 | 2.069 | 6.133 |  | 70 | 0 | 3.61 | 0.88 | 24.3 | 3.67 | 1.549 | 5.627 |  |
| 14 | 70 | 0 | 3.03 | 0.82 | 27.0 | 3.08 | 1.414 | 4.957 |  | 70 | 0 | 2.70 | 0.81 | 30.1 | 2.66 | 1.037 | 4.859 |  |
| 24 | 70 | 0 | 1.05 | 0.52 | 49.0 | 0.99 | 0.2391 | 3.354 |  | 70 | 2 | 0.79 | 0.46 | 57.7 | 0.71 | 0 | 1.843 |  |
