# Supplementary material for: A single-dose, randomized crossover study in healthy Chinese subjects to evaluate pharmacokinetics and bioequivalence of two capsules of calcium dobesilate 0.5 g under fasting and fed conditions
Source: PLoS One. 2023 Apr 21;18(4):e0284576. doi: 10.1371/journal.pone.0284576 (PMC10121042; doi:10.1371/journal.pone.0284576)
Supplement: S1 File — (DOCX) [file pone.0284576.s008.docx]

National medicine approval number: H20030088

**An open, randomized, single-dose, two-cycle crossover design was used to evaluate the bioequivalence of calcium dobesilate capsule preparation versus its reference preparation (Doxium®) in healthy Chinese subjects**

Drug name: calcium dobesilate capsule

Size: 0.5g

Scheme number: QB-20180101/CRC-C1806

Version number/Version date: V1.0/2018-03-02

Clinical trial institution: Shanghai Xuhui Central Hospital

Main researcher: Liu Yanmei / 021-54030254

Mailing address: No. 966 Middle Huaihai Road, Xuhui District, Shanghai

Analysis and testing unit: Shanghai Xuhui Central Hospital

Contact: Zhang Mengqi / 18821146094

Mailing address: No. 966 Middle Huaihai Road, Xuhui District, Shanghai

Data management unit: Shanghai Fosun Pharmaceutical Industry Development Co., LTD

Contact person: Tian Zhang / 021-33987593

Mailing address: Building A, No. 1289, Yishan Road, Shanghai

Statistical analysis unit: Shanghai Bojia Pharmaceutical Technology Co., LTD

Contact person: Zheng Qingshan / 021-20228630

Mailing Address: Room 501, Building 1, Shihe Business Center, Lane 2277, Zuchongzhi Road, Pudong New Area, Shanghai

Applicant for drug registration: Shanghai Zhaohui Pharmaceutical Co., LTD

Contact person/contact: Hu Chaoxin / 18915789957 ‬

Mailing address: 2151 Fuyuan Road, Baoshan District, Shanghai

Contract research organization: Shanghai Fosun Xingtai Pharmaceutical Technology Co., LTD

Contact person: Liu Xuejun / 13761842520

Mailing address: Building 7, 1999 Zhangheng Road, Pudong New Area, Shanghai

**Abstract**

| **Study Name** | An open, randomized, single-dose, two-cycle crossover design was used to evaluate the bioequivalence of calcium dobesilate capsule preparation versus its reference preparation (Doxium®) in healthy Chinese subjects |
| --- | --- |
| **Purpose** | Main purpose: To evaluate the bioequivalence of Calcium dobesilate capsules (0.5 g) produced by Shanghai Zhaohui Pharmaceutical Co., Ltd. and Doxium® capsules (0.5 g) produced by Ebewe Pharma Ges.M.B.H. NFG.kg in Chinese healthy subjects and to conduct in vivo bioequivalence evaluation after marketing.  Secondary objective: To observe the safety of calcium dobesilate capsule (0.5 g) and reference preparation (Doxium®, 0.5 g) in healthy subjects. |
| **Study design** | An open, randomized, single-dose, two-cycle crossover design was used in this study, which was administered in fasting and postprandial state. |
| **Subjects** | Healthy adult male or female |
| **Estimated sample size** | Fasting study in 26 cases, fed study in 72 cases. |
| **Test drug and dosage** | Test preparation: Calcium dobesilate capsule, 0.5 g per tablet, Shanghai Zhaohui Pharmaceutical Co., LTD.  Reference preparation: Calcium Doxium sulfonate capsule (Doxium®), 0.5 g per tablet, Ebewe Pharma ges.M.B.H. NFG.kg.  Usage and Dosage: take orally, 0.5 g x 1 tablet, take it with 240 mL water. |
| **Inclusion criteria** | 1) Aged 18 to 40, male or female;  2) Weight: male ≥50 kg, female ≥45 kg; Body mass index (BMI) in the range of 19-26 kg/m^2^ (including the critical value);  3) No history of diseases such as artless, liver, kidney, digestive tract, nervous system, mental and metabolic abnormalities, and no history of serious infection or serious injury;  4) The important indicators of physical examination, vital signs examination, electrocardiogram examination and laboratory examination are normal or within the acceptable range considered by the investigator;  5) During the trial period and within 3 months after the last medication, no child care plan and reliable contraceptive measures can be taken;  6) Fully understand the purpose and requirements of the trial, voluntarily participate in the clinical trial and sign a written informed consent, and be able to complete the whole trial process according to the requirements of the trial. |
| **Exclusion criteria** | 1) known allergic history, allergic disease or allergic constitution to test preparations and any of their components or related preparations;  2) Have a history of any diseases that may affect the safety of participating in the study or the in-vivo process of the tested drug, including the history of diseases of the central nervous system, cardiovascular system, digestive system, respiratory system, urinary system, blood system, immunology, psychiatry and metabolic abnormalities;  3) Blood donation or blood loss ≥400 mL within 3 months before inclusion;  4) Those who have taken any drugs within 2 weeks prior to screening;  5) Participants in clinical trials of other drugs within 3 months prior to inclusion;  6) Current or former drug user, or previous alcoholic (i.e., more than 28 standard units per week for men and 21 standard units per week for women. 1 Standard units containing 14 g alcohol, such as 360 mL beer or 45 mL 40% alcohol spirits or 150 mL wine), or drinking regularly (more than 14 standard units per week) within 3 months prior to the trial;  7) Smokers who smoke more than 10 cigarettes a day;  8) Hepatitis B surface antigen (HBsAg), HCV antibody, syphilis antibody, HIV antibody positive;  9) Positive pregnancy test (for women);  10) Positive breath tests for substance abuse (morphine, THC, methylamphetamine, dimethylene dioxy-amphetamine, ketamine and cocaine) or alcohol;  11) Abnormal and clinically significant chest X-ray examination results (anteroposterior);  12) Other factors that the investigator considers inappropriate for participation in the study. |
| **Withdraw standard** | 1) The subject withdraws the informed consent;  2) If serious adverse events occur and the investigator considers that the trial should be discontinued, the subject still enters the safety set;  3) Serious violation of the protocol. |
| **Termination standard** | 1) Serious safety problems occurred during the study;  2) Major errors in the clinical trial plan were found during the trial;  3) The drug registration applicant requests termination of the trial;  4) The ethics committee requested termination of the trial;  5) The drug administration requested termination of the test. |
| **Methods** | Subjects underwent screening tests within 28 days prior to dosing.  The fasting administration test or postprandial administration test in this study was divided into 2 periods. Each period lasted 2 days, and there was a single dose in each period. The selected subjects were assigned random numbers in screening order one day before the first administration, and the wash-out between the two periods was more than 3 days. The process of second period is the same as the first one. Only after PK blood sample collection and safety evaluation are completed on the second day of each period, subjects can leave the phase I clinical trial center.  Fasting trial: Subjects will be admitted to the phase I clinical trial center one day before each trial period, with unified life management. The subjects ate dinner at around 18:00 and supper at around 21:00, and then fasted but water was allowed. At around 08:00 on the first and fourth day of the trial, the investigators distributed test or reference preparations. Subjects took the medicine on an empty stomach with 240 mL water. Lunch and dinner were taken 4 h and 10 h after administration.  Postprandial trial: Subjects will be admitted to the phase I clinical trial center one day before each trial period and unified living management will be implemented. The subjects ate dinner at about 18:00, and then fasted but water was allowed. At around 08:00 on the first and fourth day of the trial, subjects ate a high-fat and high-calorie diet (about 800-1000 kcal calories, including about 150 kcal protein, 250 kcal carbohydrates and 500-600 kcal fat), which was eaten up within 30 minutes. 30 min after the subjects started eating, the investigators issued the test preparation or reference preparation, which was taken by subjects with 240 mL water. Lunch and dinner were taken 4 h and 10 h after administration.  Venous blood was collected at 16 time points, including 0 h (within 60 min before administration) and 1, 2, 3, 3.5, 4, 4.5, 5, 5.5, 6, 7, 8, 10, 12, 14 and 24 h after administration. 2 mL of blood was collected each time and placed in the anticoagulant collection vessels with corresponding labels of heparin sodium. The plasma was separated at 1500 g for 10 min (set temperature 4ºC) within 60 min after blood collection. Plasma samples were placed in 2 labeled sample tubes and stored immediately in a low temperature refrigerator (set temperature -80ºC) pending measurement.  Body temperature (oral), sitting blood pressure, and pulse were measured 1 day before, 0 h before (within 1 hour before), 4 h after (±0.5 h), and 24 h after (±1 h) each dose. Subjects were also required to complete physical examinations, 12-lead electrocardiograms, and laboratory examinations during the screening and at the end of the second period of fasting and postprandial study. |
| **Biological analysis** | Biological samples are analyzed by the Central Laboratory of Shanghai Xuhui Central Hospital.  Plasma calcium dobesilate was determined by liquid chromatography-tandem mass spectrometry (LC-MS/MS). |
| **Evaluation index** | Pharmacokinetic (PK) parameters evaluation indexes:  1) Main PK parameters: C_max_, AUC_0-T_, AUC_0-∞_  2) Secondary PK parameters: T_max_, T_1/2z_, λ_z_  Safety evaluation indexes:  1) Vital signs measurement: Body temperature (oral), pulse and sitting blood pressure were measured during screening, 1 day before, 0 h before (within 1 hour), 4 h after (±0.5 h) and 24 h after administration (±1 h). Acceptable range of reference values: body temperature (oral) : 35.5-37.2℃; Pulse: 50-100 beats/min; Blood pressure: 90-140 mmHg systolic and 50-90 mmHg diastolic.  2) Physical examination: the time was during the screening and at the end of fasting and postprandial study.  3) Laboratory examination: during the screening and at the end of fasting and postprandial study, including:  A) Routine blood examination, including white blood cells, red blood cells, hemoglobin, platelets, absolute values of lymphocytes, monocytes, neutrophils, eosinophils and basophils.  B) Routine urine examination, including PH, specific gravity, bilirubin, glucose, occult blood, urobilogen, protein, white blood cell and red blood cell.  C) Blood biochemical tests, including total bilirubin, alanine aminotransferase, aspartate aminotransferase, total protein, albumin, urea nitrogen, creatinine, fasting blood glucose, potassium, sodium and chlorine.  4) 12-lead electrocardiogram examination: during the screening and at the end of fasting and postprandial study;  5) Incidence of adverse events: Serious adverse events were collected from the signing of informed consent, and general adverse events and important adverse events were collected from the administration. |
| **Statistical analysis** | 1. Analysis of blood drug concentration (C) -time (T) data: Draw individual and average C-T curves; The mean, standard deviation, median, maximum, minimum and coefficient of variation of plasma drug concentration at the time point were listed. 2. PK parameter analysis: PK parameters of each subject were calculated by non-compartmental model, including C_max_, AUC_0-T_, AUC_0-∞_, T_max_, T_1/2Z,_ etc. The arithmetic mean, standard deviation, coefficient of variation, median, maximum, minimum and geometric mean of each parameter were calculated. 3. Analysis of main evaluation indicators: Linear model was used to analyze the variation model of test preparations/reference preparations. The 90% confidence interval of geometric mean ratio of the main PK parameters (C_max_, AUC) of the two drugs was calculated and their equivalence was compared. 4. Safety analysis: calculate the incidence of adverse events, adverse reactions and systematic classification. |
| **Evaluation criteria for bioequivalence** | AUC and C_max_ of the logarithmic transformed test preparation were in the range of 80.00%-125.00% of the reference preparation. |
| **Stage and time** | Screening: within 28 days before administration  Trial stage: Fasting and postprandial study were divided into 2 periods, 2 days per cycle (washout period between two doses ≥3 days) |

**1 Background**

**1.1 Basis for setting questions**

According to "Opinions of The General Office of the State Council on carrying out consistency evaluation of the quality and Efficacy of generic Drugs" (State Development Office [2016] No. 8) [1], "Opinions of The State Council on Reforming the Review and Approval System of Drugs and Medical Devices" (Guofa-2015-44) [2] "Improve the quality of generic drugs, accelerate the consistency evaluation of generic drug quality, Strive to complete the quality consistency evaluation of national Essential medicine oral preparations and reference preparations by the end of 2018 ", "Promote the quality consistency evaluation of generic drugs, and carry out the quality consistency evaluation of approved generic drugs in stages and batches according to the principle of consistency with the quality and efficacy of the original drugs. Drug manufacturers shall evaluate the consistency of quality of their products with reference preparations in accordance with the prescribed methods, and submit the evaluation results to the Administration of Food and Drug Administration. "Shanghai Zhaohui Pharmaceutical Co., Ltd. plans to evaluate the consistency of quality and efficacy of calcium dobesilate capsules on the market.

This trial was submitted by Shanghai Zhaohui Pharmaceutical Co., LTD., and commissioned by the Phase I Clinical Trial Laboratory of Drug Clinical Trial Institution, Shanghai Xuhui Central Hospital, to evaluate the post-marketing consistency of the tested preparation and the reference preparation in Chinese healthy subjects.

This clinical trial must comply with the Declaration of Helsinki, the Good Practice for Quality Management of Clinical Trials (GCP) [3] issued by the CFDA and other relevant laws and regulations. Before carrying out bioequivalence clinical trials, clinical trials can only BE carried out if they have passed the approval of the Ethics committee, signed cooperation agreements with clinical trial institutions, publicized on the information publicity platform of the State Food and Drug Administration and put on a record of BE trials [4]. Prior to the initiation of this trial, the clinical trial can only be conducted after obtaining the approval or filing of the relevant regulatory department. The trial is also subject to compliance with all applicable regulatory requirements.

**1.2 Clinical application of experimental drugs [5,6]**

**1.2.1 Mechanism of action**

Pharmacological action: As a vascular protective agent, calcium dobesilate mainly acts in the following three aspects:

1) Microvascular wall ---- increases resistance and reduces pathological hyperpermeability

2) Blood flow ---- reduces the viscosity of blood and plasma, reduces the high cohesion of platelets, and prevents thrombosis.

3) For microvascular lesions, this product eliminates or relieves clinical signs (edema, capillary oozing, lower limb heaviness, and pressure sensation).

1.2.2 Indications, usage and dosage, and applicable population

**Indications:**

1) Treatment of microvascular disease:

Diabetic microangiopathy - retinopathy and glomerulosclerosis (Kie-Weil syndrome);

Microvascular injury - with capillary fragility and increased permeability, capillary disease, and cyanosis of hands and feet.

2) For the adjuvant treatment of chronic venous insufficiency (varicose vein syndrome) and sequelae (post-embolization syndrome, leg ulcers, purpura dermatitis, and other stasis skin diseases, peripheral angiostasis edema, etc.).

**Usage and dosage**

Diabetic retinopathy: 1 capsule (0.5g) at a time, 3 times a day (morning, afternoon and evening once);

Other indications: One capsule (0.5g) at a time, twice a day (once in the morning and once in the evening). If the clinical symptoms improve, the evening medication should be omitted (about one month after the medication).

**1.2.3 Adverse reactions**

Immune system disorders: Rare: hypersensitivity (including rash, atopic dermatitis, pruritus, urticaria, facial edema); Very rare: an allergic reaction.

Neurological disorders: Common: headache.

Gastrointestinal disorders: Common: abdominal pain, diarrhea, nausea, vomiting.

Bone and connective tissue diseases: Common: arthralgia, myalgia.

Systemic disease and administration site status: Rare: fever, chills, fatigue, fatigue

Abnormal test results: Common: elevated alanine aminotransferase.

Once the drug is stopped, these reactions are reversible.

Spontaneous post-marketing reports: Diseases of the blood and lymphatic system: agranulocytosis, neutropenia, leukopenia.

**1.2.4 Precautions**

Patients with severe renal insufficiency requiring dialysis should be reduced.

**1.2.5 Taboos**

Allergic to any component of this product.

**1.3 Reference preparation selection basis**

According to CFDA Guiding Principles for Selection and Determination of Reference Preparations for Common Oral Solid Preparations (No. 61, 2016) [7], "Reference preparations are preferred to be domestically marketed original drugs", "original drugs refer to the first drugs approved to be marketed at home and abroad with complete and sufficient safety and effectiveness data as the basis for marketing", In addition, Doxium® (calcium dobesilate capsules) 0.5g produced by Ebewe Pharma Ges.M.B.H.NSfg. KG, the original developer of this product, is listed in the Circular of the State Administration on the Publication of the List of Reference Preparations for Generic Drugs (Batch 8) (No. 116, 2017).

In conclusion, Doxium® (0.5 g) was selected as the reference preparation for consistency evaluation of test preparation (0.5 g) in this study.

**1.4 Apply for specifications**

Shanghai Zhaohui Pharmaceutical Co., Ltd. has listed a variety of calcium dobesilate capsules (national medicine approval number H20330088) the specification is 0.5g, so the test drug specification is 0.5g.

**1.5 Contents of this research**

In this study, an open, randomized, single-dose, two-cycle crossover design was used. The original drug Doxium® (0.5g) was used as the reference preparation to evaluate the consistency of oral calcium dobesilate capsules (0.5g), a test preparation produced by Shanghai Zhaohui Pharmaceutical Co., Ltd. in healthy Chinese subjects.

In this study, a fasting drug administration test was conducted first, followed by postprandial drug administration test, and 26 and 72 subjects were enrolled respectively. The fasting and postprandial administration tests were divided into 2 cycles, each cycle was 2 days in total. 1 day before the first cycle of medication, selected subjects will be assigned a random number according to the screening sequence. Each subject will receive the test preparation or reference preparation respectively on a fasting or postprandial state. After a cleansing period of ≥3 days, another preparation will be cross-administered in the second cycle. Before administration, heparin indwelling needles were implanted in the superficial veins of the upper limbs of the subjects, and venous blood was collected at 16 time points 0 h before administration (within 60 min before administration) and 1, 2, 3, 3.5, 4, 4.5, 5, 5.5, 6, 7, 8, 10, 12, 14 and 24 h after administration, with 2 mL of blood collected each time.

The evaluation indexes of this experiment were pharmacokinetic parameters (AUC0-t, AUC0-∞, Cmax, etc.), and the safety indexes were evaluated through complaints of discomfort, vital signs examination, physical examination, laboratory examination, 12-lead electrocardiogram examination, etc. Adverse events (AE) and serious adverse events (SAEs) were evaluated according to NCI-CTC AE 4.03, medically coded according to MedDRA 20.0 (or above), disaggregated by SOC/PT, and should be described by a system of occurrence, severity, frequency, frequency, and incidence. Serious adverse events require a detailed list.

The statistical unit of this experiment used SAS (9.3 or higher) to randomly generate random tables according to 1:1 block. Each subject was randomly assigned to receive the test or reference preparation using block randomization. The random table is reproducible, and the initial seed parameter of the random number set will be saved.

**2 Experimental design**

**2.1 Overall design of this study**

According to the Four General Principles 9011 of Chinese Pharmacopoeia (2015 edition), Guiding Principles for Human Bioavailability and Bioequivalence Testing of Pharmaceutical Preparations [8], Technical Guidelines for Human Bioavailability and Bioequivalence Research of Chemical Pharmaceutical Preparations [9], and Technical guidelines for human Bioequivalence Research of chemical generic drugs with pharmacokinetic parameters as the endpoint evaluation index As required by "(Circular No. 61 of 2016) [10] et al., the clinical study of bioequivalence for consistency evaluation of calcium dobesilate capsules adopted an open, randomized, single-dose, two-cycle crossover trial design, including fasting and postmeal administration.

In this study, the fasting drug administration test or postprandial drug administration test was divided into 2 cycles, each cycle was 2 days in total. One day before the first cycle of drug administration, the selected subjects were assigned random numbers according to the screening sequence. Each subject would receive the test preparation or reference preparation respectively in the fasting or postprandial state, and after a cleaning period of ≥3 days, another preparation would be cross-administered in the second cycle.

**2.2 Pharmacokinetic characteristics [6]**

After oral administration of 500 mg calcium dobesilate, the serum concentration level was above 6 μg/mL during the 3rd and 10th hours, and the maximum serum concentration (C maximum) was 8 μg/mL on average 6 hours later (t maximum). The blood concentration was about 3 μg/mL 24 hours after administration. The protein binding rate is 20-25%. Animal studies have shown that calcium dobesilate does not cross the blood-brain barrier or placental barrier, but whether the same is true in humans is unclear. It can be found in breast milk in trace amounts (0.4 μg/mL was observed in one study after 1500 mg administration).

Calcium dobesilate does not enter enterohepatic circulation and is excreted mainly in its original form, with only 10% excreted as metabolites. Within 24 hours of administration, approximately 50% of the oral dose is excreted in the urine and approximately 50% in the stool.

The plasma half-life is about 5 hours.

**2.2.1 Pharmacokinetic Characteristics of special populations**

It is not clear to what extent renal dysfunction affects the pharmacokinetic properties of calcium dobesilate.

Since it is not clear whether calcium dobesilate crosses the human placental barrier, pregnant women should weigh the pros and cons of using this drug.

Calcium dobesilate can be found in breast milk in trace amounts (0.4 μg/mL in breast milk after 1500 mg administration). As a precaution, lactating women should stop taking medication or breastfeeding.

**2.3 Previous Research**

According to the research report of Huang Lu et al. [12], the bioequivalence of calcium dobesilate. disperse tablets and calcium dobesilate tablets were evaluated. A randomized crossover design with two preparations and two cycles was used in 19 male healthy volunteers. The statistical test showed that the dispersible tablets were bioequivalent to the reference tablets.

Previous studies have shown that food significantly affects the process of calcium dobesilate. in vivo, delaying drug absorption in healthy volunteers, and reducing plasma drug concentration and bioavailability [13].

**2.4 Detailed scheme design**

In accordance with the principles of an open, random, single administration, and two-cycle crossover, a fasting drug administration test was conducted first, followed by a postprandial drug administration test, and selected subjects were assigned random numbers in the order of screening 1 day before the first cycle of drug administration. After fasting for ≥10 hours 1 day prior to the first cycle, each subject will receive either the test preparation or the reference preparation, and have blood collected according to the following schedule. After the ≥ 3-day cleansing period, another preparation will be cross-administered in the second cycle.

Blood collection time points: Venous blood was collected at 16-time points 0 h before administration (within 60 min before administration) and 1, 2, 3, 3.5, 4, 4.5, 5, 5.5, 6, 7, 8, 10, 12, 14 and 24 h after administration.

Enrolled subjects were randomly assigned to groups A, B, C, or D to receive the test preparation or reference preparation on a fasting or postprandial state, respectively. The test procedure table is as follows:

| Sequence | Period 1  (Day 1-2) | Wash-out  (≥3 days) | Period 2  (Day 4-5) |
| --- | --- | --- | --- |
| A (C) | T | - | R |
| B (D) | R | - | T |

Note: 1. T is the test preparation, R is the reference preparation;

2. Drug administration on an empty stomach and postprandial state:

Fasting administration: Subjects were randomly divided into Group A and Group B:

Group A (fasting): subject preparation - washout period - reference preparation,

Group B (fasting): reference preparation - washout period - test preparation,

Postprandial administration: Subjects were randomly divided into Group C and Group D:

Group C (after meal): test preparation - washout period - reference preparation,

Group D (after meal): reference preparation - washout period - test preparation;

3. Fasting or postprandial administration tests were evenly divided: the first period (day 1-2), the wash-out (≥3 days), and the second period (day 4-5). Subjects were administered on day 1 and day 4, respectively.

Subjects will be assigned unique screening numbers S001, S002, S003... And so on. Subjects will be assigned random numbers 1 day before the first cycle of administration according to the order of screening. Fasting trial random numbers are R01, R02, R03... R26 in turn, the randomized numbers of postprandial administration trials were R27, R28, R29... By analogy, and according to the randomization table, two preparations were respectively administered in the fasting or postprandial state.

Enrolled subjects were admitted to the Phase I clinical trial ward 1 day before each cycle of medication. They ate a light diet in the evening and fasted for more than 10 h overnight before medication. In the morning of the next day, 240mL water was used to take the test drug on an empty stomach or postprandial state. Blood samples were collected 16 times (2 mL/ time) before and 24 h after each administration to determine the blood concentration of calcium dobesilate to assess its bioequivalence.

Subjects will be allowed to leave the Phase I clinical study unit on day 2 of each cycle after completing blood sample collection and safety assessment. Subjects' body temperature (oral), sitting blood pressure, and pulse were measured 1 day before administration, 0 h before administration (within 1 hour before administration), 4 h after administration (±0.5 h), and 24 h after administration (±1 h). Subjects also need to complete a physical examination, 12-lead electrocardiogram examination, and laboratory examination at the end of the screening period, fasting administration test, and postprandial administration test.

The test procedure and PK blood collection time window are shown in Table 1 and Table 2 respectively.

**Table 1. Bioequivalence trial flow table of calcium dobesilate capsules**

| **Program** | **Screening period** | **Day -1** | **First period (2 days), Day 1 before and after administration (h)** | | | | | | | **Leave hospital ^5^** | **Wash-out (≥3天)** | **For the second period (2 days), repeat the operation on day 1** | | | | | | | **Leave hospital** |
| --- | --- | --- | --- | --- | --- | --- | --- | --- | --- | --- | --- | --- | --- | --- | --- | --- | --- | --- | --- |
|  |  |  | **Day 1-2** | | | | | | | **Day 2** | **Day 3** | **Day 4-5** | | | | | | | **Day 5** |
|  |  |  | **0 h** | **1 h** | **2 h** | **3 h** | **3.5 h** | **4 h** | **4.5-24 h** | **24 h** | **-** | **0 h** | **1 h** | **2 h** | **3 h** | **3.5 h** | **4 h** | **4.5-24 h** | **24 h** |
| Medical history | **×** | **×** |  |  |  |  |  |  |  |  |  |  |  |  |  |  |  |  |  |
| Physical examination | **×** |  |  |  |  |  |  |  |  |  |  |  |  |  |  |  |  |  | **×** |
| Inclusion and exclusion criteria | **×** | **×^8^** |  |  |  |  |  |  |  |  |  |  |  |  |  |  |  |  |  |
| Informed consent | **×** |  |  |  |  |  |  |  |  |  |  |  |  |  |  |  |  |  |  |
| Assignment filter number | **×** |  |  |  |  |  |  |  |  |  |  |  |  |  |  |  |  |  |  |
| Randomization |  | **×** |  |  |  |  |  |  |  |  |  |  |  |  |  |  |  |  |  |
| Admitted to Phase I ward |  | **×** |  |  |  |  |  |  |  |  |  |  |  |  |  |  |  |  |  |
| Body weight | **×** |  |  |  |  |  |  |  |  |  |  |  |  |  |  |  |  |  |  |
| Height | **×** |  |  |  |  |  |  |  |  |  |  |  |  |  |  |  |  |  |  |
| Virological examination **^1^** | **×** |  |  |  |  |  |  |  |  |  |  |  |  |  |  |  |  |  |  |
| Fasting |  | **×** |  |  |  |  |  |  |  |  | **×** |  |  |  |  |  |  |  |  |
| Administration |  |  | **×** |  |  |  |  |  |  |  |  | **×** |  |  |  |  |  |  |  |
| Dining |  |  | **×^4^** |  |  |  |  | **×** |  |  |  | **×** |  |  |  |  | **×** |  |  |
| 12-lead electrocardiogram | **×** | **×^3^** |  |  |  |  |  |  |  |  |  |  |  |  |  |  |  |  | **×** |
| Vital sign ^2^ | **×** | **×** | **×** |  |  |  |  | **×** |  | **×** | **×** | **×** |  |  |  |  | **×** |  | **×** |
| Blood routine | **×** | **×^3^** |  |  |  |  |  |  |  |  |  |  |  |  |  |  |  |  | **×** |
| Blood biochemistry | **×** | **×^3^** |  |  |  |  |  |  |  |  |  |  |  |  |  |  |  |  | **×** |
| Urine routine | **×** | **×^3^** |  |  |  |  |  |  |  |  |  |  |  |  |  |  |  |  | **×** |
| Urine pregnancy |  | **×** |  |  |  |  |  |  |  |  | **×** |  |  |  |  |  |  |  |  |
| Blood pregnancy | **×** |  |  |  |  |  |  |  |  |  |  |  |  |  |  |  |  |  |  |
| Drug abuse |  | **×^8^** |  |  |  |  |  |  |  |  |  |  |  |  |  |  |  |  |  |
| Alcohol breath test |  | **×^8^** |  |  |  |  |  |  |  |  |  |  |  |  |  |  |  |  |  |
| Chest X-ray | **×^6^** | |  |  |  |  |  |  |  |  |  |  |  |  |  |  |  |  |  |
| PK sampling**^7^** |  |  | **×** | **×** | **×** | **×** | **×** | **×** | **×** |  |  | **×** | **×** | **×** | **×** | **×** | **×** | **×** |  |
| Leave hospital |  |  |  |  |  |  |  |  |  | **×** |  |  |  |  |  |  |  |  | **×** |
| Drug combination | **×** | **×** | **×** | **×** | **×** | **×** | **×** | **×** | **×** | **×** | **×** | **×** | **×** | **×** | **×** | **×** | **×** | **×** | **×** |
| Compliance evaluation | **×** | **×** | **×** | **×** | **×** | **×** | **×** | **×** | **×** | **×** | **×** | **×** | **×** | **×** | **×** | **×** | **×** | **×** | **×** |
| Adverse event | **×** | **×** | **×** | **×** | **×** | **×** | **×** | **×** | **×** | **×** | **×** | **×** | **×** | **×** | **×** | **×** | **×** | **×** | **×** |

1. Virological examination: including hepatitis B surface antigen (HBsAg), hepatitis C virus antibody (HCV-Ab), human immunodeficiency virus antibody (HIV-Ab), and syphilis antibody;

2. Vital signs: Body temperature (oral), pulse, and sitting blood pressure were measured during the screening period, the day before each administration, 0 h before each administration (within 1 hour before each administration), 4 h after administration (±0.5 h) and 24 h after administration (±1 h), respectively;

3. Blood routine, blood biochemistry, urine routine, and 12-lead electrocardiogram: 1 day before the first dose. If the dose is administered within 14 days after the screening period test, no retest is required before the dose (day -1), and a retest is required after 14 days;

4. Postprandial test Subjects in groups C and D began to eat high-fat and high-heat meals 30 minutes before administration on the test day (caloric composition: About 800-1000 kcal, including protein about 150 kcal, carbohydrate about 250 kcal, fat about 500-600 kcal), and eat within 30 minutes, 30 minutes after eating time to take the test drug; Group A and Group B subjects are not applicable;

5. The patient can be discharged 24 hours after the first cycle of administration, according to the actual situation of the experiment;

6. It can be checked on the 1st day;

7. Blood sampling points 4.5-24 h included: 4.5, 5, 5.5, 6, 7, 8, 10, 12, 14, and 24 h;

8. One day before the first dose.

**Table 2. PK blood collection time window**

| **Time point** | **Time window** |
| --- | --- |
| 0h | Within 1 hour before the administration |
| 1h | ±2min |
| 2h | ±2min |
| 3h | ±2min |
| 3.5h | ±2min |
| 4h | ±5min |
| 4.5h | ±5min |
| 5h | ±5min |
| 5.5h | ±5min |
| 6h | ±5min |
| 7h | ±5min |
| 8h | ±5min |
| 10h | ±10min |
| 12h | ±10min |
| 14h | ±10min |
| 24h | ±10min |

**3 Subject Selection**

**3.1 Inclusion Criteria of Subjects (all subjects are eligible for inclusion)**

1) 18 ~40 years old, male or female;

2) Weight: male ≥50 kg, female ≥45 kg; Body mass index (BMI) in the range of 19-26 kg/m^2^ (including critical value);

3) Centerlessness, liver, kidney, digestive tract, nervous system, mental and metabolic disorders, no history of serious infection and serious injury;

4) Physical examination, vital signs examination, electrocardiogram examination, and laboratory examination were all normal or within the range considered acceptable by the researcher;

5) No parenting plan and reliable contraceptive measures were available during the trial period and within 3 months after the last dose;

6) Fully understand the purpose and requirements of the trial, voluntarily participate in the clinical trial, sign the written informed consent, and be able to complete the whole process of the trial as required.

**3.2 Exclusion Criteria for Subjects (if one item is satisfied, exclusion is achieved)**

1) Known allergic history, allergic disease or allergic constitution to the test preparation and any component or related preparation;

2) A history of any medical conditions, including central nervous system, cardiovascular system, digestive system, respiratory system, urinary system, blood system, immunology, psychiatric and metabolic disorders, that may affect the subject's safety in participating in the study or the in-vivo process of the test drug;

3) Blood donation or blood loss ≥400 mL in the 3 months before inclusion;

4) Patients who had taken any medication within 2 weeks before screening;

5) Participants who had participated in other drug clinical trials within 3 months before inclusion;

6) Current or former drug users or previous heavy drinkers (i.e., more than 28 standard units per week for men and 21 standard units per week for women). 1 standard unit containing 14 grams of alcohol, such as 360 mL beer or 45 mL 40% spirits or 150 mL wine), or regular alcohol consumption (more than 14 standard units per week) in the 3 months prior to the trial;

7) Smokers who smoke more than 10 cigarettes a day;

8) Hepatitis B surface antigen (HbsAg), HCV antibody, syphilis antibody, HIV antibody positive;

9) Positive pregnancy test (applicable to female);

10) Positive results of drug abuse tests (morphine, tetrahydrocannabinol, methylamphetamine, dimethylene dioxyamphetamine, ketamine and cocaine) or alcohol breath tests;

11) Abnormal chest X-ray (posterior and anterior) examination results with clinical significance;

12) There are other factors that the researcher considers unsuitable for participating in the experiment.

**3.3 Subject shedding Criteria (Shedding shall occur if meeting any of the following criteria)**

1) the subject withdraws the informed consent;

2) If serious adverse events occur and the study is considered to be discontinued, the subject remains in the safety set;

3) Serious violation of the scheme.

**3.4 Test termination criteria (The test will be terminated if it meets any of the following criteria)**

1) Serious safety problems occur in the test;

2) Major errors in the clinical trial protocol are found in the trial;

3) The drug registration applicant requests the termination of the trial;

4) The ethics committee requests the test to be terminated;

5) The drug regulatory department requires the test to be terminated.

3.5 Treatment principles for subject shedding

For shedding subjects, their original medical records should be kept and analyzed:

1) For the subject who has fallen off, the researcher should contact the subject as far as possible to inquire about the reason and possible safety; Subjects who shed before the first dose do not need to undergo follow-up examinations out of the group. Subjects who shed after dose should complete follow-up examinations out of the group (physical examination, vital signs examination, 12-lead ECG examination, laboratory examination) whenever possible.

2) For subjects who withdraw from the study due to adverse events, the investigator should take appropriate treatment measures according to the actual situation of the subjects, and follow up until the adverse events have improved or returned to normal/abnormal clinical significance or baseline state or the subjects lose follow-up;

3) All shedding subjects should keep relevant test data properly.

3.6 Taboos and Restrictions

1) The subject shall not use any drug other than the test drug unless it is required for a medical emergency within 14 days prior to administration or during the course of the study without the prior consent of the principal investigator or sponsor;

2) Fasting for at least 10 h before administration, 4 h after administration, and 1 h before and after administration; Keep the upper body upright for 4 h after taking the medicine;

3) The subjects are not allowed to smoke or eat any food or drink containing caffeine, alcohol, or grapefruit during the test;

4) The subjects must take medicine, collect blood and undergo a physical examination on time during the test, and avoid strenuous physical activities;

5) No parenting plan and reliable contraceptive use during the trial period and within 3 months after the last dose.

**4 Trial process**

**4.1 Test drug information**

The experimental drugs were all provided by Shanghai Zhaohui Pharmaceutical Co., LTD., and detailed information is shown in Table 3:

**Table 3 Information of experimental drugs**

| **Drug information** | Test preparation: calcium dobesilate capsules | Test preparation: calcium dobesilate capsules (Trade name：Doxium^®^) |
| --- | --- | --- |
| **Dosage form/specification** | Capsule/ 0.5 g | Capsule/ 0.5 g |
| **Preservation condition** | Keep it sealed in a dry place, cool and dark (no more than 20℃ from light) | Store in a cool and dry place, sealed away from light |
| **Route of administration** | Oral administration | Oral administration |
| **Manufacture** | Shanghai Zhaohui Pharmaceutical Co., LTD | Ebewe Pharma Ges.m.b.H. Nfg.KG |
| **Drug packaging specification and quantity** | 2*10 capsules/board/box, aluminum foil, polyvinyl chloride solid medicinal hard sheet packaging | 20 grains/box, aluminum plastic plate packing |

**4.2 Receipt, distribution and storage of experimental drugs**

The investigator must ensure that all investigational drugs are used only for participants in the clinical trial, and that the dosage and use of investigational drugs are in accordance with the trial protocol. No investigational drugs shall be given to any non-participants in the clinical trial.

The sponsor shall provide clinical trial institutions and investigators with a sufficient quantity of investigational drugs. The clinical trial institutions and investigators shall randomly select the drugs for clinical trial and the drugs for retention sample, which shall be in the same batch as the drugs for trial. The quantity of retained samples shall meet the requirement of five full tests according to the quality standard.

The sponsor and investigator entrust an eligible independent third party to retain samples of the investigational drug until 2 years after the drug is approved for marketing.

Test drugs should be stored according to drug storage conditions. The drug delivery form shall be signed by two persons in duplicate, with the clinical research unit and the sponsor holding one copy each. After the study, the remaining drugs and empty boxes were stored together with the retained samples.

The monitor is responsible for monitoring the transportation, supply, use, storage of investigational drugs and the disposal of surplus drugs.

**4.3 Study the determination of administration dose**

When conducting bioavailability and bioequivalence studies of drug preparations in accordance with the Guiding Principles for Human Bioavailability and Bioequivalence Testing [8], the dose administered should generally be consistent with the clinical single dose, and should not exceed the clinical recommended single maximum dose or the proven safe dose. The test preparation and the reference preparation should generally be given equal doses. According to the marketing instructions of the test preparation calcium dobesilate capsules and reference preparation, the test preparation 0.5g x 1 capsule was administered orally for a single time in fasting or postprandial state, respectively, and was compared with the reference preparation 0.5g x 1 capsule. Therefore, the dose was designed to be 0.5 g.

**4.4 Subject administration method**

**4.4.1 Random Grouping Method**

The order in which subjects receive the test preparation or the reference preparation is determined by a random table. Randomized tables for fasting and postprandial administration trials will be prepared separately. Random tables are randomly generated by SAS (9.3 or later) in 1:1 area groups. Fasting test and postprandial test were randomized.

After signing the informed consent, subjects will be given a "screening number" to participate in the screening physical examination, which will be randomized 1 day before the fasting or the first cycle of the postprandial test. Eligible subjects will be given a random number according to the order of screening.

In the analysis phase, researchers involved in sample detection and analysis should keep the analysis of biological samples blind, that is, researchers involved in sample analysis will be unaware of the random table.

The randomization table will be kept uniformly under controlled conditions. The investigator is responsible for ensuring compliance randomization tables to conduct the study.

Any subject who withdraws or is withdrawn from a randomized clinical trial after taking a study drug will be classified as a dropped subject, their randomization number will be retained and they will not be allowed to re-enter the trial.

Subjects who are unable to continue to participate in the study after randomization, fasting administration or postprandial administration for special reasons before taking the study drug will be replaced by subjects who are medically cleared before the study but not enrolled. The replacement subjects will be assigned a subject random number that changes from R to T in the dropped subject random number (e.g., Subject T06 will replace Subject R06 and subject T27 will replace subject R27). The replacement subject receives the same grouping sequence as the shedding subject.

**4.4.2 Administration Instructions**

Subjects will be required to maintain an upright upper body and avoid bed for 4 hours after taking the medication, unless there is a clinical requirement to change their position or any physical need.

Fasting administration trial: Subjects will be admitted to the Phase I clinical trial research ward one day before each cycle of trial, unified living management, and no food or drink other than uniform diet is allowed. The subjects ate dinner at around 18:00 and midnight snack at around 21:00, and then abated from drinking water. At around 08:00 on the first and fourth day of the experiment, the researchers distributed the test preparation or reference preparation. The subjects took the medicine on an empty stomach and took it with 240 mL water. Eat lunch and dinner at 4 h and 10 h after taking the medicine.

Postprandial drug administration trial: Subjects were admitted to the Phase I clinical trial research ward one day before each cycle of trial, unified life management, and no food or drink other than uniform diet was allowed. The subjects ate dinner at around 18:00 and then abstained from water. At around 08:00 on day 1 and day 4 of the experiment (calculated according to the 3-day cleansing period), subjects ate high-fat and high-calorie meals (about 800-1000 kcal calories, including 150 kcal protein, 250 kcal carbohydrate and 500-600 kcal fat). It is usually made up of two slices of bread, one beef Patty, one scrambled egg, 250mL milk in a carton, two hash browns, 10 grams of cheese, and 10 grams of butter, which may be adjusted according to the caloric changes of certain foods during the trial.), finished eating within 30 minutes; Thirty minutes after the subjects began to eat, the study preparation or reference preparation was given by the researchers and taken with 240 mL water. Eat lunch and dinner at 4 h and 10 h after taking the medicine.

**4.5 Other therapeutic agents before and during the trial**

During this trial period, any drug other than those used to treat adverse events is prohibited.

Any drug other than the test drug used during the trial shall be recorded in detail in the original record and case report form, which shall include the name of the drug, dosage, date of administration and indications for use.

**4.6 Medication compliance**

In the recruitment and screening stage, the purpose of the trial, basic information of the test drug, research scheme, test process, administration scheme (such as dosage, administration method, cycle, etc.), clinical observation, frequency and process of biological sample collection, potential risks of participating in the trial, subsidies and compensation are introduced in detail, so as to make the subjects fully informed and willing to participate, and improve the medication compliance. Before dosing, carefully check subject number, dosing randomization list, dosing dosage and order; After administration, count the remaining amount of the test drug and the empty package, and immediately check the subject's hands and mouth (oral medication) to ensure that the drug was taken by the subject.

**4.7 Clinical observation and follow-up**

Prior to the start of the study, healthy subjects must carefully read and sign an informed consent form approved by the Ethics Committee. Carry out the inspection and test procedures according to the study schedule (Table 1).

**4.7.1 Screening period**

Unless otherwise noted, the following screening steps must be performed after informed consent has been signed and should be completed within 4 weeks prior to initiation of investigational drug administration:

1) Assign screening number and collect demographic data: gender, date of birth, nationality, height, weight, etc.;

2) Daily or weekly cigarette, alcohol or drug use (number of daily or weekly drinks; The number of cigarettes smoked daily; Drug use);

3) Physical examination, medical history inquiry, drug combination and non-drug treatment;

4) Measurement of vital signs and clinical laboratory tests (blood routine, urine routine, blood biochemistry, virology), 12-lead electrocardiogram, pregnancy test (blood, female) and other indicators.

After screening, subjects who meet the inclusion criteria can be preliminarily enrolled in this test. After preliminary screening, chest X-ray (posterior and anterior) examination is performed to rule out pulmonary disease. If the subject can present the results of chest X-ray examination within 3 months prior to the signing date of the informed consent, or the results of virology test within 1 month prior to the signing date of the informed consent, the test results will be considered valid and the Research Center will not retest the subject.

**4.7.2 One day before administration**

Enrolled subjects were admitted to the Phase I clinical trial ward 1 day before the first cycle of administration. After admission, they were asked again about their medical history, drug combination and non-drug treatment. Confirm compliance with discharge standards; Sitting blood pressure, pulse, temperature (oral), alcohol and drug abuse (morphine, tetrahydrocannabinol, methylamphetamine, dimethylene dioxyamphetamine, ketamine and cocaine), The blood routine, blood biochemistry, urine routine, 12-lead electrocardiogram, chest X-ray (posterior and anterior position) and other tests were checked again to see whether they were in line with the inclusion standard or within the acceptable range considered by the researcher. Female subjects will have to undergo a urine pregnancy test and those with a negative result will be enrolled. (Note: Blood routine, blood biochemistry, urine routine and 12-lead electrocardiogram do not need to be retested before administration if the drug is administered within 14 days after the test; Retest after 14 days.)

Participants were randomly assigned to groups A, B, C, or D to receive the subject or reference formulations on an fasting or postprandial basis 1 day before the first cycle of the fasting or postprandial dosing trials. Eat a light diet at night and fast for more than 10 hours overnight before taking the medicine.

**4.7.3 Administration observation period**

On the morning of the administration, 240mL water was used to take the test drug on an empty stomach or after meal. Water was prohibited for 1 h before and after the administration, food was fast for 4 h after the administration, and standard meals were taken at 4 h and 10 h after the administration. Heparin indwelling needles were placed in the superficial veins of the upper limbs of the subjects before administration, and venous blood was collected at 16 time points 0 h before administration (within 60 min before administration) and 1, 2, 3, 3.5, 4, 4.5, 5, 5.5, 6, 7, 8, 10, 12, 14 and 24 h after administration, with 2 mL of blood collected each time. The samples were placed in labeled heparin sodium anticoagulant collection vessels, gently mixed upside-down, centrifuged at 1500 g for 10 minutes (4ºC, set temperature) within 60 minutes after blood collection, and the plasma was separated after centrifugation. The plasma samples were placed in two labeled sample tubes and immediately stored in a cryogenic refrigerator (-80ºC, set temperature) for testing.

Close observation was required after administration. Sitting blood pressure, pulse, and body temperature (oral) were measured at 4 h (±0.5 h) and 24 h (±1 h) after administration. Physical examination, 12-lead electrocardiogram examination, and clinical laboratory examination were performed at the end of fasting or postprandial administration test (day 2 of the second cycle test). During the trial, adverse event records, safety evaluation, drug combination and non-drug treatment, compliance evaluation, etc.

**4.7.4 Clinical observation indicators**

4.7.4.1 Measurement of vital signs: Body temperature (oral), pulse and sitting blood pressure were measured during the screening period, the day before each administration, 0 h before each administration (within 1 hour before administration), 4 h after administration (±0.5 h) and 24 h after administration (±1 h), respectively. Acceptable range of reference values: Body temperature (oral cavity): 35.5-37.2ºC; Pulse: 50-100 times/min; Blood pressure: Systolic blood pressure is 90-140 mmHg, diastolic blood pressure is 50-90 mmHg.

A physical examination is also required during the screening period and at the end of the fasting or postprandial administration test.

4.7.4.2 Laboratory examination and electrocardiogram examination

The following tests should be performed during the screening period and at the end of the fasting or postprandial administration test:

1) Blood routine examination, including the absolute values of white blood cells, red blood cells, hemoglobin, platelets, lymphocytes, monocytes, neutrophils, eosinophils and basophils.

2) Urine routine examination, including PH, specific gravity, bilirubin, glucose, occult blood, urobilogen, protein, white blood cell, red blood cell.

3) Blood biochemical test, including total bilirubin, alanine aminotransferase, aspartate aminotransferase, total protein, albumin, urea nitrogen, creatinine, fasting blood glucose, potassium, sodium, chlorine.

4) Virological examination: Hepatitis B surface antigen (HBsAg), hepatitis C virus antibody (HCV-Ab), human immunodeficiency virus antibody (HIV-Ab), syphilis antibody, only during the screening period.

5) 12-lead electrocardiogram of the subject was recorded.

4.7.4.3 Other examinations: chest X-ray examination (conducted before drug administration in the first cycle after qualified preliminary screening); Pregnancy test (for women, blood pregnancy test during screening period, urine pregnancy test 1 day before each dosing); Alcohol breath test and drug abuse screening (1 day before initial dosing).

**4.7.5 Follow-up**

In case of unresolved adverse events, subjects should be followed up until symptoms, signs, or test indicators return to normal/abnormal clinical significance or baseline status or subjects are lost to follow-up. If necessary, the investigator may order a review or other examination as required by the safety assessment.

**5. Sample collection, transportation and storage**

Intravenous indwelling needle was used to collect blood samples from the superficial veins of the upper limbs of the subjects, and 2 mL of blood was collected each time into the vacuum collection vessel containing heparin sodium anticoagulant. The permissible deviation of the collection time point after drug administration was shown in Table 2 below item 2.4.

Blood sample collection and disposal shall be carried out temporarily as described in the above scheme (Section 4.7.3). If special operations such as avoiding light are required for blood sample collection and disposal according to the results of exploration or verification of biological sample detection and analysis methods, operations shall be carried out according to the relevant operation manuals and other materials provided by the biological sample analysis and detection unit.

Subject number, test period and blood collection point number should be indicated on the biological sample collection tubes and plasma sample storage tubes, e.g. Crc-c1806-rzs: The test number is CRC-C1806; r is the random number of the subject (3 digits); z is the test cycle, A is the first cycle of fasting drug administration test, B is the second cycle of fasting drug administration test, C is the first cycle of postprandial drug administration test, D is the second cycle of postprandial drug administration test. s is the sampling point number, starting from 01 and increasing gradually. The numbering information of the biological sample collection tube and the plasma sample storage tube should be consistent. The positive sample number of the plasma sample storage tube is CRC-C1806-rzs, and the backup sample number is CRC-C1806B-rzs (B indicates backup).

**6 Drug concentration detection**

**6.1 Sampling time point**

Pharmacokinetic blood samples were collected at a total of 16 time points 0 h before administration (within 60 min before administration) and 1, 2, 3, 3.5, 4, 4.5, 5, 5.5, 6, 7, 8, 10, 12, 14 and 24 h after administration per cycle

**6.2 Method Verification**

The blood samples were analyzed by the Central Laboratory of Shanghai Xuhui District Central Hospital. For hydroxyl benzene sulfonic acid calcium determination of plasma concentration, according to a report in the reference [11], and plasma samples by ion pair extraction high performance liquid chromatography (HPLC) method (ion pairing extraction and HPLC) determination of hydroxyl benzene sulfonic acid calcium blood drug concentration in 0.1 ~ 50  g/mL and good linear relationship between peak area, Detection limit of 0.1  g/mL, and through the methodology validation, can be used in the human body hydroxyl benzene sulfonic acid calcium determination of blood drug concentration and dosage of human pharmacokinetic studies.

This laboratory will adopt liquid chromatoc-tandem mass spectrometry (LC-MS/MS) for determination of plasma calcium hydroxybenzenesulfate. In order to ensure the reliability of the analytical method of LC-MS/MS for determination of plasma calcium, complete methodological verification must be carried out according to the structure of the substance to be tested, biological matrix and expected concentration range. To establish a sensitive, specific, accurate and reliable method for quantitative analysis of biological samples. The whole analysis process should follow the laboratory SOP and relevant national guidelines.

The content of methodology verification can be seen in the analysis and testing plan of the biological sample analysis and testing unit.

In order to investigate the stability of whole blood sample collection, it may be necessary to take whole blood samples (about 1 mL for each subject) from 6 fasting subjects 5 h after administration, and investigate according to the analytical test protocol.

**6.3 Determination of calcium hydroxybenzene sulfonate concentration in the sample to be tested**

Quality control must be carried out during the determination of drug concentration in the sample to ensure the reliability of the established method in practical application. Each unknown sample is generally measured once, and can be retested if necessary. The standard curve and quality control samples of high, medium and low concentrations should be accompanied with the measurement of each batch of biological samples. At least two samples per concentration and should be evenly distributed in the unknown sample test sequence. When the number of unknown samples in an analysis batch is large, the number of quality control samples of each concentration should be increased so that the number of quality control samples is greater than 5% of the total number of unknown samples. The deviation of the test results of quality control samples should generally be less than 15%, and the results of 1/3 QC samples with a maximum concentration of 50% of each QC sample are allowed to exceed the limit. If the concentration of the sample is higher than the upper limit of quantification, it should be diluted with a blank substrate and re-determined. Select at least 10% of the sample for reanalysis. Reanalysis samples selected near Cmax and elimination phase samples collected, at least 67% of the samples should conform to the sample deviation between ±20%.

7. Pharmacokinetic parameters and safety evaluation indexes

7.1 Pharmacokinetic parameters for evaluation

PK parameters were analyzed and calculated using non-atrioventricular model, and the main evaluation indexes were Cmax, AUC0-t and AUC0-∞.

| **Parameters** | **Interpretation** |
| --- | --- |
| C_max_ | Peak concentration. It was directly obtained from the measured data of blood concentration and time. |
| AUC_0-t_ | The area under the curve from zero to the time at which the lowest blood concentration can be detected. Calculated by linear ladder rule: AUC_(i, i+1)_=(Ti+ 1-ti)(Ci+Ci+1)/2, AUC0-t is the sum of all AUC_(i, i+1)_. |
| AUC_0-∞_ | The area under the curve extrapolated from zero to infinity. AUC0-∞=AUC0-t+Ct/λz(Ct is the last determinable blood concentration). |
| T_max_ | Peak time. It was directly obtained from the measured data of blood concentration and time. |
| t_1/2z_ | Terminal elimination half-life. T_1/2z_=ln2/λ_z_。 |
| λ_z_ | The slope of the final section of the semi-logarithmic time curve is calculated using linear regression method, eliminating the rate constant. |
| AUC__%Extrap_ | Percentage of residual area. AUC__%Extrap_=[(AUC_0-∞_-AUC_0-t_)/AUC_0-∞_]×100% |

**7.2 Safety evaluation index**

The safety indexes were evaluated by complaints of discomfort, vital signs examination, laboratory examination, electrocardiogram examination, etc. Measurement of vital signs: Body temperature (oral), pulse and sitting blood pressure were measured during screening period, the day before each administration, before each administration (0 h) (within 1 h before administration), 4 h (±0.5 h) after administration and 24 h (±1 h) after administration, respectively. Clinical laboratory examination (blood routine examination, urine routine examination, blood biochemical examination), 12-lead electrocardiogram examination and physical examination were conducted at the end of the screening period and the second cycle of fasting and postprandial drug administration test.

Adverse events (AE) and serious adverse events (SAE) were evaluated according to NCI-CTC AE 4.03, medically coded according to MedDRA 20.0 (or above), and disaggregated by SOC/PT. The analysis of adverse events should be described according to the system, severity, frequency, frequency and incidence of the events, and the relationship with the test drug list. Laboratory test results describe what is normal before the test but abnormal after taking the drug and how the abnormal changes relate to the test drug. Adverse events, serious adverse events need to be detailed list.

**7.3 Adverse Events and serious adverse events**

**7.3.1 Definition of Adverse Events**

An adverse event (AE) is an adverse medical event that occurs after a patient or clinical trial subject receives a drug, but is not necessarily causally related to the treatment. An AE can be any adverse and unexpected sign (including abnormal laboratory test values), symptom, or temporary disease state, and does not have to have a clear causal relationship with the study drug.

An adverse drug reaction (ADR) is an adverse event associated with a tested drug at any dose. All adverse events that the investigator or sponsor determines have a reasonable causal relationship with the investigational drug are adverse drug reactions.

**7.3.2 Definition of Serious Adverse Events**

Serious adverse events (SAEs) refer to medical events during clinical trials that require hospitalization or prolong hospitalization, cause disability, affect the ability to work, threaten life or death, or result in congenital malformations, including the following unexpected medical events:

Events leading to death;

Life-threatening events (defined as subjects at risk of death at the time of the event);

Events requiring hospitalization or prolonged hospitalization;

Events that cause permanent or severe disability/disability/affect the ability to work;

A congenital abnormality or birth defect.

**7.3.3 Reports of adverse events**

Adverse events include general adverse events, important adverse events and serious adverse events. In this study, serious adverse events were collected from the signing of the informed consent, general adverse events and important adverse events were collected from the administration of the drug until any adverse medical events occurred at the end of the trial, regardless of whether there was a causal relationship with the test drug, were judged as adverse events.

Any adverse events, regardless of their severity or whether they are related to the study, are recorded and described in the Adverse Event Form. The investigator shall record in detail any adverse events that occurred to the subjects, including: description of the adverse events and all related symptoms, occurrence time, severity, cause of the adverse events, correlation with the test drug, duration, actions taken, and final results and outcomes.

**7.3.4 Report of serious adverse events**

According to the Drug Registration Administration Measures and relevant regulations of China's GCP, if any SAE occurs during the study, the investigator should report it to the Ethics Committee of Shanghai Xuhui Central Hospital, the applicant for Drug Registration (Shanghai Zhaohui Pharmaceutical Co., LTD.), Shanghai Food and Drug Administration and the State Food and Drug Administration within 24 hours after being informed of it. Contact information for the report is shown in Table 4.

**Table 4 Contacts for serious adverse event reports**

| **Organization** | **contact** | **fax/phone/address** |
| --- | --- | --- |
| Shanghai Xuhui Central Hospital | Ethics committee  Sages of Europe and America | Phone: 021-54043676  Fax: 021-54043676 |
| Shanghai Xuhui Central Hospital | Drug clinical trial facility  Zhang Meiwei | Phone: 021-54030254  Fax: 021-54030254 |
| Shanghai Zhaohui Pharmaceutical Co., LTD | Hu Zhaoxin | Phone: 18915789957 |
| China Food and Drug Administration | Department of Pharmaceutical Research Supervision, Department of Pharmaceutical and Cosmetic Registration Administration | Phone: 010-88330797  Fax: 010-88363228  Address: Building 2, Yard 26, Xuanwumen West Street, Xicheng District, Beijing  Zip code: 100053 |
| Shanghai Food and Drug Administration | Drugs and Cosmetics Registry | Phone: 021-23118187  Fax: 021-63558718 |
| Shanghai Municipal Health and Family Planning Commission | Medical administration Department | Phone: 021-83090065  ylaqsae@163.com |
| National Health and Family Planning Commission | Medical Safety and Blood Division, Medical Administration Hospital Authority | Phone: 010-68792734 |

The sponsor is responsible for monitoring the entire study implementation process and will ensure that the Center completes all SAE reports in compliance with regulatory and local regulatory requirements. The sponsor will report the SAE to the appropriate regulatory authority as required by the regulatory authority and local regulations.

**7.3.5 Severity of adverse events**

When filling out the adverse event form, researchers will use common adverse event evaluation criteria (NCI-CTCAE version 4.03) to determine the intensity of adverse events. For a unified standard, AE event intensity is classified as follows:

Level 1: mild, asymptomatic or mild; Only clinically or clinically seen; No treatment required.

Level 2: moderate, requiring minor, local, or non-invasive treatment; Age-appropriate limitation in instrumental activities of daily living *.

Level 3: serious or medically important but not immediately life-threatening; Resulting in hospitalization or prolonged hospitalization; To cripple; Limited personal activities of daily living **.

Level 4: life-threatening; Urgent treatment is required.

Level 5: Death related to AE.

Level 3 and 4 are classified as severe.

Note: Activities of Daily Living (ADL)

* : Instrumental activities of daily living refer to cooking, buying daily supplies or clothes, making telephone calls, managing money and goods, etc.

** : Personal activities of daily living refer to bathing, dressing/undressing, eating, using the toilet, and taking medicine, excluding being bedridden.

**7.3.6 Assessment of causality (5-point method)**

The causal relationship between AE and the tested drug was determined by the following criteria:

It is definitely related: the occurrence of AE and the use of the test drug have a reasonable time sequence. AE is the known adverse reaction of the test drug, which is alleviated or disappeared after withdrawal of the drug, and repeated after re-use of the drug, which cannot be explained by the subject's own disease.

It is likely to be related: the occurrence of AE and the use of the test drug have a reasonable chronological sequence, AE is a known adverse effect of the test drug, the response alleviates or disappears after discontinuation of the test drug and cannot be explained by the subject's own disease, and the effect after re-use is unknown.

It may be relevant: the occurrence of AE and the use of the test drug have a reasonable chronological sequence, AE is a known or suspected adverse reaction to the test drug, however, there are other factors that may cause the event, such as disease, drug combination, etc.; The effects of drug discontinuation are unclear, unclear or lacking conclusive information.

May not be relevant: The occurrence of AE and the use of the test drug have a reasonable chronological sequence, but the event is not of a known type of adverse drug reaction and is most likely caused by the subject's disease or other treatment.

Certainly not: the occurrence of AE and the administration of the test drug have no reasonable chronological sequence, such as events that occurred before the use of the test drug; Is not a known adverse drug reaction; Or AE is caused by other factors, such as the subject's disease, other treatments, or combination of medications.

The adverse reactions of test drugs were "definitely related", "probably related" and "probably related", and the incidence rate of adverse reactions was calculated according to the combined results.

**7.3.7 Measures Taken**

In the event of AE occurring throughout the clinical trial, the investigator may take necessary management measures according to the subject's situation, such as: no measures taken, study dose adjustment or temporary discontinuation of study medication, study medication combined, and not applicable. In the presence of SAE, the investigator must immediately take necessary action to protect the subject's safety. All aes should be recorded in detail and the results should be followed up until the subject's symptoms, signs, or test indicators return to normal/abnormal clinical significance or baseline status or the subject is lost to follow-up. According to the severity of adverse events, researchers can choose a variety of follow-up methods such as inpatient, outpatient, home visit, and communication.

**7.3.8 Outcome of adverse events**

1) Recovery: Complete recovery, or medical or surgical treatment, to the level at which subjects performed their first trial-related activities after signing the informed consent form.

2) Improvement: The condition is improving and the subject is expected to recover. This term may only be used for subjects who have completed the trial.

3) Persistence: Adverse events occur that cause significant and permanent disability/disability (e.g., blindness, deafness, and paralysis). Any adverse event with residual sequelae should be considered as a serious adverse event.

4) Aggravation: aggravation of adverse events and deterioration of the condition.

5) Death: Subject dies due to adverse events.

6) Unknown: This term is only used for subjects who have lost follow-up.

**7.3.9 Treatment and report of pregnancy events in the test**

If the subject (or the subject's sexual partner) experiences a pregnancy event during the study period, the investigator shall immediately discontinue the study and discontinue the study drug. The investigator should communicate scientifically and rigorously with the subject based on medication information, inform her/him of the possible effects and risks of the study medication to the pregnant woman and the fetus, and leave it up to the subject to decide whether to terminate or continue the pregnancy.

Within 24 hours of confirming the subject's (or the subject's sexual partner's) occurrence of pregnancy, the investigator shall complete the initial pregnancy report form and report it to the sponsor and the hospital Ethics Committee.

If the subject (or the subject's sexual partner) decides to terminate the pregnancy, the serious adverse event report form shall be completed by the investigator within 24 hours after the termination is known and reported to the sponsor, the hospital ethics committee, and the State Food and Drug Administration.

If the subject (or the subject's sexual partner) decides to continue the pregnancy, the pregnancy will be followed up every 3 months until the subject (or the subject's sexual partner) has delivered and the outcome of the pregnancy is known. Within 24 hours of each pregnancy follow-up, the investigator completed the pregnancy report and reported it to the sponsor and the hospital ethics committee.

**8 Data Management**

**8.1 Data Collection**

In this study, the subject data will be filled in the designated paper CRF, and the first copy will be sent to the data manager for two-person and double-entry work to enter the data into the project database system. The carbon copy will be retained as an investigator copy for the investigator.

Management of clinical data will be performed in accordance with Institute Standards for Applicable Clinical Data Exchange Standards and data cleansing procedures to ensure data integrity and accuracy, such as removal of errors and inconsistent data.

Research Center staff should be responsible for filling out the CRF. For all subjects who sign the ICF, the researcher or authorized staff shall carefully record the items in the CRF in detail, with no blank items or missing items (blank Spaces should be filled in according to the actual situation); All data in the CRF must be checked against the subject's original data to ensure accuracy.

The investigator shall attach the original test sheet or a copy to the subject's study medical record; Abnormal laboratory or test data should be verified by the investigator and whether they are clinically significant; Researchers should follow the CRF filling guidelines strictly.

**8.2 Locking the Database**

When the following conditions are met, you can lock data.

 all data are input database;

 all questions are resolved;

 analysis has been defined and judge the crowd.

The locked data file will not be modified without the authorization of the sponsor.

The data management process is described in detail in the Data Management Plan (DMP).

**9 Statistical Analysis**

**9.1 Population Analysis**

Safety data set (SS): all randomized/substitute, and accepted the study drug, has a record of safety index of the subjects.

Full analysis set (FAS): all randomized/substitute, and accept that the subjects of study drugs.

PK concentrations set (PKCS): all randomized/substitute, and accepted the study drug, during the test at least one effective blood drug concentration data of the subjects.

PK parameter set (PKPS): all randomized/backup and accepted the study drug, at least one valid PK parameters during the period of test subjects. Those who do not include PKPS include: a) those who seriously violate the scheme selection, affect the PK parameter results, or cannot estimate the parameters; b) subject's concentration before administration &gt; 5% of Cmax; c) Drug combination occurred during the test and had an impact on PK parameters; d) Subjects who developed vomiting during 2 times the median Tmax of the study drug; e) The first sample was Cmax, and no subject data was collected for the early sample (5-15 minutes after administration).

BE set (BES): to complete the drug test on an empty stomach or after meal to two cycles, and both cycle into PKPS subjects.

**9.2 Analysis Content**

**9.2.1 Subject distribution**

 listed subjects distribution analysis of the data set.

 list participants list data set.

**9.2.2 Demographic Data and baseline analysis**

 according to FAS is analyzed.

 descriptive statistics demographic data and other baseline characteristics.

 continuous variable calculated the number of cases, the mean, standard deviation, median, minimum and maximum.

 data to calculate frequency and frequency count and level.

**9.2.3 Analysis of medication compliance and drug combination**

 according to FAS is analyzed.

 medication adherence: analysis whether volume using experimental drug on time.

 adopt list form a detailed description of drug combination.

 according to ATC classification combined drug use frequency.

9.2.4 Pharmacokinetic analysis

 key PK data points agreed: a) sampling time point is not beyond the time window, sampling time calculation as planned; b) If the time window is exceeded, the actual sampling time shall be calculated. c) All blood drug concentration values below the lower limit of quantification were treated as "0" before Tmax during PK analysis and as deletion after Tmax. However, in the descriptive statistical analysis, 0 was treated and the number of BQL at each time point was indicated.

 blood drug concentration (c), time (t) data analysis: individual and average c - t curve drawing, half logarithm curve of c - t; The mean, standard deviation, median, maximum, minimum and coefficient of variation of drug concentration at time point were listed.

 analysis: PK parameters calculated by the atrioventricular model subjects medicine generation parameters. Arithmetic mean, standard deviation, coefficient of variation, median, maximum, minimum and geometric mean of each parameter were calculated at the same time. Descriptive statistical analysis was not performed for AUC0-∞, t1/2z and AUC_%Extrap if subjects AUC_%Extrap > 20%.

 the main evaluation index analysis: the BES, Cmax, AUC_0 - t_ and AUC_0 -∞_ up after logarithmic transformation to carry on the analysis of variance (ANOVA). In the analysis of variance model, order, drug and period are used as fixed effects, while subjects (order) are used as random effects. The 90% confidence interval of the geometric mean ratio of the main indicators (test preparation/reference preparation) was calculated, if it was within the equivalent interval (80.00%-125.00%), it was judged as bioequivalent. The results of two unilateral t tests are also listed. At the same time, the coefficient of variation in individual parameters was calculated. If AUC__%Extrap_ > 20% of subjects, AUC_0-∞_ will not be included in the analysis.

**9.2.5 Security Analysis**

 according to the analysis of SS.

 adverse events according to the example of international medical terminology dictionary (MedDRA20.0 or above) coding.

 calculation of adverse events, serious adverse events/reaction, lead to fall off the incidence of adverse events/response.

 list/reaction, serious adverse events, adverse events, lead to loss of adverse events/reaction of SOC and PT cases and cases, calculate the incidence.

 all kinds of adverse event/reaction, serious adverse events, leading to loss of adverse events/reaction cases detailed list.

 laboratory index, electrocardiogram (ecg) after test abnormal turn "normal" or "abnormal increase of the number of cases.

 listed laboratory index, electrocardiogram (ecg), medical and clinical interpretation.

**9.3 Analyze the software and general requirements**

 PK parameters are calculated by the Phoenix WinNonlin7.0 (or later), the other using SAS 9.4 software analysis (or later versions).

 detailed statistical methods will be provided in the statistical analysis plan.

 no interim analysis in this study.

**9.4 Sample Size estimation**

In this study, a double-cross design was adopted, with pharmacokinetic parameters (AUC, Cmax) as the main analysis indexes. In the fasting test, unilateral α=0.05, β=0.2, intra-CV 20% (reference to Intra variation in pretest), the ratio between test preparation and reference preparation was 0.93-1.07, and the bioequivalent interval was 80.00%-125.00%, calculated by PASS (Version 11.0.7) software. The test sample size was 24 cases, and the fasting test sample size was set at 26 cases considering the shedding condition. In postpranpranal tests, unilateral α=0.05, β=0.2, Intra-CV 25%, ratio of test preparation to reference preparation 0.90-1.07, bioequivalence interval 80.00%-125.00%, and PASS (Version 11.0.7) software were used to calculate the estimated sample size of 62 cases. The sample size for postprandial tests was set at 72 cases, taking into account shedding.

That is, 26 subjects were to be included in the fasting test and 72 subjects in the postprandial test.

**10 Quality Assurance**

Sponsors, investigators and CROs shall establish their own quality assurance systems, perform their respective responsibilities, and strictly follow the clinical trial protocol and adopt the appropriate standard operating procedures to ensure the quality control of clinical trials and the implementation of the quality assurance system.

**10.1 Quality assurance of the clinical trial process**

Before starting clinical trials, researchers should receive training on the trial protocol, so that they can fully understand and know the specific connotation of the clinical trial protocol and its indicators. The sponsor shall verify the basic conditions of the clinical trial to ensure that the clinical trial conditions can meet the requirements of the program. During the trial, the investigator shall carefully perform clinical operations and other work in accordance with the SOP and the requirements of the trial protocol, and record them in a true, timely, complete and standardized manner. Quality control personnel shall check the test process and corresponding original records. After the end of the test, the research unit shall sort out the corresponding project files, and archive them after checking. The quality assurance department of the clinical research unit shall conduct an implementability audit of the tests conducted. When non-conforming items are found, the researchers should be informed to correct in time, and the correction situation should be tracked.

**10.2 Quality assurance of sample testing process**

The testing laboratory undertaking biological analysis shall establish a quality assurance system, carry out quality verification in strict accordance with the relevant domestic and international technical guidelines, laboratory standard operating procedures and quality control procedures, formulate a verification plan, and carry out verification in accordance with the content of the plan. The content of verification includes and is not limited to: personnel training and authorization, sample management, standard product management, instrument and equipment verification, calibration and maintenance, methodology confirmation, sample testing, data verification, etc. According to the progress of the test work and the verification results of the quality control personnel, the laboratory quality assurance personnel shall carry out the inspection of the analysis and test process and test results in different categories.

**10.3 Quality assurance of data transfer, calculation and reporting processes**

After researchers input data into CRF, quality control personnel should check the consistency of CRF data and original records to ensure that data is accurately input into CRF. The monitor 100% verifies that the test data entry in the CRF system is complete, accurate, and consistent with the original medical records. Query data items that are in doubt or inconsistent with the original medical records in time. Data entry clerks and researchers are urged to answer questions, check and correct inconsistent data.

The personnel of the data management department shall verify the quality of data entry by means of logical verification, and send the results of doubts to the researchers in the form of doubts, who will verify and modify them. Quality control personnel check data management files and database data.

The sponsor shall, according to the needs and in combination with the progress of the trial and the verification results of the quality control personnel/inspectors, conduct an inspection in different areas of the above clinical trial process, sample testing process, data, report and calculation process.

**10.4 Risk assessment and risk management**

A single dose of 0.5 g was used in this clinical trial. After each cycle of administration, medical staff shall continuously and closely observe the possible adverse events of the subjects. The drug is well tolerated even after long-term administration. If there are occasional stomach discomfort, nausea, heartburn, loss of appetite and other symptoms, the dosage should be reduced as appropriate, and the administration should be stopped if necessary. Since this was a bioequivalence test, two capsules were given in two cycles of fasting or postprandial administration, and the dosage was small. However, in order to avoid adverse reactions, researchers still need to pay close attention and take appropriate medical measures if necessary.

In the event of adverse events occurring during the subject's participation in the study, the relevant treatment costs and/or subsidies (if applicable) shall first be paid by the Sponsor's insurance purchased for the study or, if not covered by the insurance, by the Sponsor.

**10.5 Solution Deviation or Violation**

All requirements specified in the research programme must be strictly implemented. Any intentional or unintentional deviation from or violation of the test protocol and the principles of the GCP may be classified as deviation from the protocol or violation of the protocol. In the process of supervision, if deviation from the scheme is found by the supervisor, the researcher or the supervisor shall fill in the deviation record, record the time of discovery, the time and process of the occurrence of the event, the reason and the corresponding treatment measures in detail, sign by the researcher, and inform the ethics committee and the sponsor. In statistical analysis reports and summary reports, analyze and report the impact of any deviations or violations on the final data and conclusions.

Evaluation should be conducted when serious protocol violations occur. If necessary, the sponsor may terminate the study in advance.

**11. Handle abnormal situations during the test**

If there is any unplanned abnormal situation during the test, it should be handled in accordance with the principle of "subject safety first, truth-seeking and pragmatic, scientific and normative", timely and detailed records should be recorded, and the impact on the test results should be assessed.

**12. Research reports and data preservation**

After the study, all parties involved in the study shall write corresponding research reports according to the research results, including methodological confirmation report, sample test report, data management report, data statistical analysis report and summary report. The format and content, attachments and maps of the report are in accordance with the requirements of the latest draft of CTD format of bioequivalence study issued by CFDA. The report shall come into force after being sorted out, reviewed, signed and sealed.

To ensure the evaluation and supervision of clinical studies by the State Food and Drug Administration and the sponsor, researchers should agree to keep all study data, including original records of subjects' hospitalization, informed consent forms, case reports, detailed records of drug distribution, etc. Paper and electronic documents generated by research units, analysis and testing units, data management and statistical analysis units shall be properly stored to ensure timely traceability. The investigator shall keep the data until 5 years after the end of the clinical trial, and the sponsor shall contact the research unit for further preservation, destruction or transfer to a third party within 6 months before the expiration of the 5 years. The sponsor shall keep the clinical trial data for at least 2 years after the approved marketing of the tested product. Except for the requirements of the State Food and Drug Administration and other regulatory authorities, without the written consent of the sponsor, the researcher shall not provide in any form to a third party.

**13 Description of Plan revision**

After the proposal is approved by the Ethics Committee, if it is to be modified, the proposal modification instructions shall be prepared and signed by the principal researcher and the sponsor. After modification of the plan, it can be implemented only after review by the Ethics Committee or filing.

**14 References**

[1] Opinions of The General Office of the State Council on Carrying out Consistent Evaluation of the Quality and Efficacy of Generic Drugs (2016) No.8.

[2] Opinions of The State Council on Reforming the Review and Approval System of Drugs and Medical Devices (Guofa-2015-44).

[3]. Good Practice for Quality Control of Drug Clinical Trials (GCP).

[4]. Announcement of the State Food and Drug Administration on the Administration of Filing Bioequivalence Tests of Chemical Drugs (No. 257, 2015).

[5]. Drug instructions for Calcium Dobesilate Capsules (Shanghai Zhaohui Pharmaceutical Co., LTD.) -- Revised: January 25, 2011.

[6]. Doxium® Drug Package Insert (Ebewe Pharma Ges.m.b.H. Nfg.KG) -- Revised: 06/07/2016.

[7] Guiding Principles for Selection and Determination of Reference Preparations for Common Oral Solid Preparations (No. 61, 2016).

[8]. Chinese Pharmacopoeia (2015 edition) Four-part General Principles 9011, Guiding Principles for Human Bioavailability and Bioequivalence Tests of Pharmaceutical Preparations

[9]. Technical Guidelines for Research on Bioavailability and Bioequivalence of Chemical Drug Preparations in Humans

[10]. Technical Guidelines for Human Bioequivalence Studies of Chemical Generic Drugs with pharmacokinetic Parameters as the end point Evaluation Index (Circular No. 61 of 2016)

[11] Rona K, Ary K. Determination of calcium dobesilate in human plasma using ion-pairing extraction and high-performance liquid chromatography.[J]. Journal of Chromatography B Biomedical Sciences & Applications, 2001, 755(1-2):245.

[12]. Huang Lu, Ge Miaomiao, Shu Chengren, et al. Bioequivalence of calcium dobesilate dispersible tablets [J]. Chinese Journal of Hospital Pharmacy, 2010, 30(11):931-933. (in Chinese)

[13] Miao Liyan, Zhang Hua, Qian Meiying, et al., Effects of calcium dobesilate capsules on pharmacokinetics in healthy people, Chinese Journal of New Medicine and Clinical Medicine, 2007 (26), 3, 196-199.
